# Essential Genetic Interactors of *SIR2* Required for Spatial Sequestration and Asymmetrical Inheritance of Protein Aggregates

**DOI:** 10.1371/journal.pgen.1004539

**Published:** 2014-07-31

**Authors:** Jia Song, Qian Yang, Junsheng Yang, Lisa Larsson, Xinxin Hao, Xuefeng Zhu, Sandra Malmgren-Hill, Marija Cvijovic, Julia Fernandez-Rodriguez, Julie Grantham, Claes M. Gustafsson, Beidong Liu, Thomas Nyström

**Affiliations:** 1 School of Life Science and Technology, Harbin Institute of Technology, Harbin, China; 2 Department of Chemistry and Molecular Biology, University of Gothenburg, Göteborg, Sweden; 3 Department of Medical Biochemistry and Cell Biology, University of Gothenburg, Göteborg, Sweden; 4 Mathematical Sciences, University of Gothenburg, Gothenburg, Sweden; 5 Mathematical Sciences, Chalmers University of Technology, Gothenburg, Sweden; 6 Centre for Cellular Imaging, University of Gothenburg, Göteborg, Sweden; The University of Arizona, United States of America

## Abstract

Sir2 is a central regulator of yeast aging and its deficiency increases daughter cell inheritance of stress- and aging-induced misfolded proteins deposited in aggregates and inclusion bodies. Here, by quantifying traits predicted to affect aggregate inheritance in a passive manner, we found that a passive diffusion model cannot explain Sir2-dependent failures in mother-biased segregation of either the small aggregates formed by the misfolded Huntingtin, Htt103Q, disease protein or heat-induced Hsp104-associated aggregates. Instead, we found that the genetic interaction network of *SIR2* comprises specific essential genes required for mother-biased segregation including those encoding components of the actin cytoskeleton, the actin-associated myosin V motor protein Myo2, and the actin organization protein calmodulin, Cmd1. Co-staining with Hsp104-GFP demonstrated that misfolded Htt103Q is sequestered into small aggregates, akin to stress foci formed upon heat stress, that fail to coalesce into inclusion bodies. Importantly, these Htt103Q foci, as well as the ATPase-defective Hsp104^Y662A^-associated structures previously shown to be stable stress foci, co-localized with Cmd1 and Myo2-enriched structures and super-resolution 3-D microscopy demonstrated that they are associated with actin cables. Moreover, we found that Hsp42 is required for formation of heat-induced Hsp104^Y662A^ foci but not Htt103Q foci suggesting that the routes employed for foci formation are not identical. In addition to genes involved in actin-dependent processes, *SIR2*-interactors required for asymmetrical inheritance of Htt103Q and heat-induced aggregates encode essential *sec* genes involved in ER-to-Golgi trafficking/ER homeostasis.

## Author Summary

Asymmetric cell division is key to cellular rejuvenation and budding yeast exploits this mode of cytokinesis to generate a young daughter cell from a mother cell that with each division grows progressively older. Thus, age physiognomies are reset in the progeny during division, a phenomenon that requires a mother-biased segregation of cytoplasmic ‘aging factors’, including damaged/aggregated proteins. There are two models for how aggregated proteins are segregating in a mother cell-biased fashion; one holds that asymmetric inheritance is a purely passive outcome of the aggregates' random but slow diffusion whereas the other model reasons that specific factors/organelles prevent free diffusion of aggregates into the daughter cell. In the present work, we tested whether the passive diffusion model or the factor-dependent model appear most relevant in explaining asymmetrical inheritance by quantifying traits predicted to affect inheritance by passive diffusion and identifying factors required for asymmetrical inheritance amongst essential genes interacting with *SIR2*; a gene shown previously to be required for mother-biased segregation. We show that passive diffusion of aggregates is not sufficient to establish mother-biased segregation and that ER to Golgi trafficking, in addition to the actin cytoskeleton, calmodulin, and the Myo2 motor protein, are key components restricting the inheritance of both heat stressed-induced aggregates and aggregates formed of the Huntington disease protein Htt103Q.

## Introduction

Cell division in budding yeast, *Saccharomyces cerevisiae*, and specific adult stem/progenitor cells includes asymmetrical inheritance of oxidized proteins, ensuring low levels of cytosolic damage in a specific cell lineage [1]–[3]. In both yeast and adult precursor cells, the lineage inheriting less damage display a longer life expectancy [1]–[3]. Thus, these singular division events provide a tractable model for how age physiognomies are reset in the progeny, which might provide clues towards therapeutically halting, or even reversing, senescence and tissue decline

In budding yeast, the control of aggregate inheritance encompasses an Hsp104-dependent retention of damaged/aggregated proteins in the mother cell [4], [5], a spatial protein quality control (SQC) that relies also on the deposition of aggregates into specific protein inclusions called Insoluble Protein Deposit (IPOD) and JUxta Nuclear Quality control compartment (JUNQ) [6]–[8]. Besides the protein remodeling factor Hsp104, the yeast gerontogene Sir2 [9]–[11] is required for asymmetrical segregation of oxidized and aggregated proteins [1], [4], [12], [13]. The role of both Hsp104 and Sir2 in establishing damage asymmetry has been linked to actin cable-dependent processes and the polarisome [5], [14]; a complex at the tip of the daughter cell required for actin cable nucleation [15], [16]. Actin cables are suggested to play a role in aggregate retention due to their (and prions') physical association with the actin cytoskeleton preventing their free diffusion into the daughter [5], [14], [17]–[19]. Sir2 deficiency reduces actin cable abundance, cytoskeletal functions, and the velocity of retrograde actin flow from the polarisome region [4], [14], [20]. This link between Sir2 and actin cable functions are consistent with data demonstrating that Sir2 affects the rate of actin folding by modulating the activity of the chaperonin CCT [14]. Actin-cables and the small heat shock protein Hsp42 are also required for the formation of peripheral aggregates [21]. Based on such results, it has been suggested that asymmetrical segregation of damaged proteins is a factor-dependent, genetically determined process, which results in the association of aggregates with structures/organelles limiting their inheritance into the daughter cell [1], [4]–[6], [14], [19].

This view is contrasting that of Li and colleagues [21], which, based on aggregate tracking experiments and modeling, argues that asymmetric inheritance is a predictable, and purely passive, outcome of aggregates' slow, random diffusion and the geometry of yeast cells. In this view, aggregate inheritance is dictated solely by the diameter of the bud neck and for how long this neck is open (generation time) for diffusion of aggregates. However, there is a large and unexplained amount of diversity in the supposedly random movement of aggregates in the aggregate population recorded by Zhou et al., [22] such that many aggregates appears stationary in the mother cell while others move in a ballistic fashion. Thus, the usefulness of employing an average diffusion coefficient for this diverse population of aggregate movements in attempting to draw conclusions about inheritance being factor dependent or purely passive has been questioned [6]. In addition, it was shown that the large aggregates in the Zhou et al., [22] study is IPOD and JUNQ inclusions that cannot diffuse freely, or randomly, since they are tethered to the vacuole and nucleus, respectively [6].

In the present work, we tested whether the passive diffusion model or the factor-dependent tethering model appear most relevant for our understanding of asymmetrical inheritance of aggregates and the asymmetry defects observed in cells lacking Sir2. To do so, we analyzed the inheritance of two reporters; the spontaneously misfolding and aggregating Huntingtin Htt103Q protein and heat-induced, Hsp104-associated aggregates and quantified the traits of *sir2* mutant cells predicted to affect the inheritance of such aggregates in a passive manner. In addition, we identified hitherto unknown factors required for asymmetrical inheritance among essential genes displaying synthetic genetic interactions with *SIR2*, in order to determine if inheritance defects is linked to specific biological processes/components or governed by passive traits. The data obtained suggest that slow and passive diffusion is not sufficient for establishing the mother-biased segregation displayed by wild type yeast cells. Instead, we found that the essential actin-associated myosin V motor protein Myo2 and the actin organization protein calmodulin, Cmd1, are required for asymmetrical inheritance and that both Htt103Q foci and heat-induced Hsp104-associated stress foci/peripheral aggregates co-localize with Myo2/Cmd1-enriched structures. Super-resolution 3-D structured illumination microscopy further showed that both Htt103Q and Hsp104 foci co-localize with actin cables. In addition, the data suggest that a fully functional ER-Golgi trafficking/ER homeostasis activity is required for restricting aggregate inheritance during yeast cytokinesis.

## Results

### Aggregate inheritance defects in *sir2*Δ cells cannot be explained by alterations in mother-daughter geometry, aggregate abundance, or generation times

**Figure 1:**
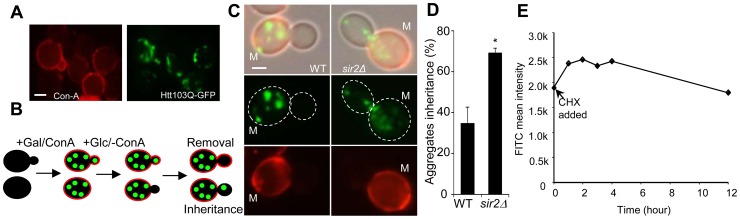
Sir2 is required for efficient mother-biased segregation of the Huntington disease model protein HttQ103. **A.** Representive images of wild type cells, stained with concanavalin A (left panel), showing HttQ103-GFP aggregates (right panel) after turning off HttQ103-GFP expression. Scale bar  = 5 µm. **B.** Schematic outline of the experimental design for segregation of HttQ103-GFP. HttQ103-GFP aggregation was triggered by inducing the *P_GAL_-HTT103Q* gene by galactose. Expression was subsequently turned off by switching the carbon source to glucose and the segregation of aggregates was scored during the next budding event. Mother cells were stained briefly with concanavalin A (red circle in the picture) allowing easy detection of new buds (not stained). **C.** Pictures of wild-type (left panel) and *sir2*Δ (right panel) cells displaying new budding events after turning off HttQ103-GFP aggregate production. Pictures from upper to lower panel are merged images, HttQ103-GFP and ConA-Alexa Fluor 647. Scale bars  = 2 µm. M =  mother cells. **D.** Quantification of aggregate inheritance in wild type and *sir2*Δ cells. All new budding events in which the mother cell displayed aggregates were quantified. E. Stability of Htt103Q aggregates analyzed by flow cytometry. Stability is measured as mean signal intensity from Htt103Q-GFP aggregates as a function of time after inhibition of protein synthesis. Data are represented as mean + s.d. An unpaired two-tailed *t-*test confirms a statistically significant difference between the two strains (*P,0.05).

For obtaining empirical, quantitative, datasets on aggregate inheritance, we used both heat-induced aggregate formation detected by Hsp104-GFP and the aggregation-prone Huntington's disease protein Htt103Q-GFP (detailed information of this construct can be found in Wang et al. 2007 [22]), which, in contrast to heat-induced aggregates, forms small and stable aggregates rather than large IPOD/JUNQ inclusions (Figure 1A; [23]–[25]). Reduced inheritance (e.g. by aggregate retention in mother cells) and aggregate removal (e.g. by disaggregation or retrograde aggregate movement in daughter cells) [14], [19] are the two processes required for establishing asymmetric aggregate distribution. Figure 1B shows a schematic illustration of how these two processes can be distinguished experimentally. Upon *HTT103Q* induction (leading to Htt103Q aggregation) by the addition of galactose, cells are stained with a fluorescent conA (concanavalinA) conjugate, which binds to glycoproteins in the cell wall. During the subsequent addition of glucose, which represses further *HTT103Q* expression, conA is washed away. This protocol enables discrimination between daughter cells present during induction of *HTT103Q* expression and aggregate formation (stained with conA), and cells generated after turning off synthesis of the aggregating protein (not stained with conA) that can only display aggregates if they (or possibly small aggregation nucleation particles) have been inherited from the mother cell (Figure 1B). Analyzing the inheritance of all visible Htt103Q foci demonstrated that wild type yeast mother cells retained Htt103Q aggregates in a quantitatively similar way as heat-induced aggregates [14], [21] during cytokinesis (Figure 1C&D) and that the absence of Sir2 reduced this retention capacity about 2-fold (Figure 1C; p = 0.02). During the time frame of the experiment, we found little or no clearance of the Htt103Q protein in conA-stained daughter cells (Figure 1E). Thus, establishment of asymmetrical aggregate distribution of both small aggregation-prone disease proteins and indigenous heat-induced Hsp104-associated inclusion bodies [6], [14] are dependent on Sir2 and involves aggregate retention in mother cells.

**Figure 2:**
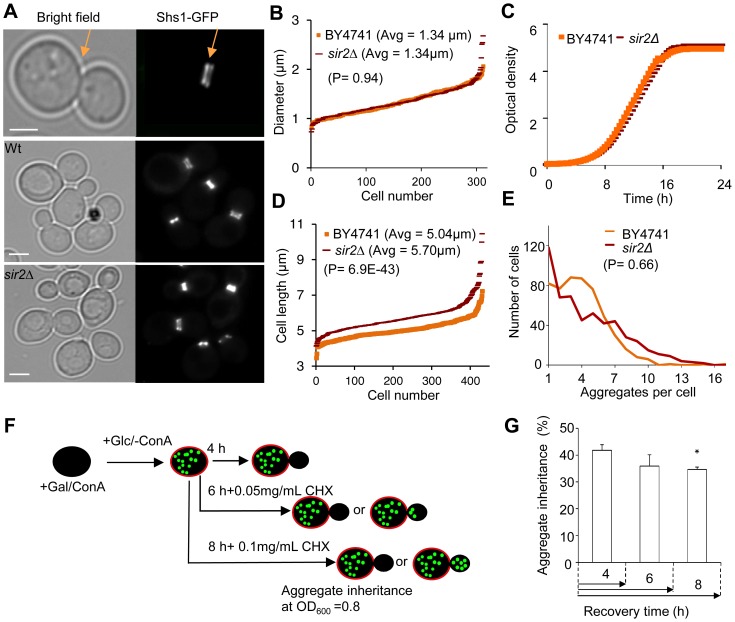
Increased inheritance of aggregates in *sir2* daughter cells cannot be explained by changes in geometrical parameters and generation time. A. Representative pictures of bud neck visualization using Shs1-Gfp as a reporter in the wild type and *sir2*Δ mutant (yellow arrows). Scale bars  = 5 µm. B. Distribution of bud neck diameters in a population of wild type (orange) and *sir2*Δ mutant (red) cells. C. Growth of *sir2*Δ (red) compared to wild type (orange) cell cultures in liquid YPD media. The generation times are depicted in the figure. (Data are represented as mean of triplicates.). D. Distribution of average mother cell length in a population of *sir2*Δ (red) and wild type (orange) cells. E. Distribution in the number of protein aggregates (Htt103Q-GFP) per cell in wild type (orange) and *sir2*Δ (red) populations. P-value is calculated from around 500 cells. The statistical significance of observed differences was determined with the two-tailed U-test (P = 0.66) demonstrating that the difference between wild type and *sir2* mutant cells is not significant. F. Schematic outline of the experimental design for the aggregate retention assay in which wild type cells are treated with low concentrations of cycloheximide (CHX) to prolong generation times. G. Inheritance of aggregates after prolonging the generation time more than two-fold using CHX at concentrations indicated. Aggregate inheritance data are represented as mean + s.d. of triplicate samples. Statistically significant differences from wild types are determined by unpaired two-tailed *t*-test. Asterisks denote significant differences between samples: *P,0.05.

Simulations suggest [21] that to allow for the 2-fold increased inheritance the bud neck between the mother and daughter has to be enlarged by a factor of 2.2–3.0 provided the aggregates move by random walk [21] and that the generation time and aggregate number is similar in the wild type and mutant cells. Using the septin ring component Shs1-Gfp as a reporter for the bud neck, we found no evidence that the mean and median bud neck diameter in wild type and *sir2*Δ mutant cells was different (Figure 2A&B). In addition, the generation time of Sir2-deficient cells was not significantly longer than that of wild type cells (Figure 2C). Moreover, the average length of a *sir2*Δ mutant mother cell is longer than a wild type mother cell (Figure 2D), which would mean that the average aggregate in a *sir2*Δ mother have to embark on a longer journey to reach the daughter, which would yield a more pronounced asymmetry in Sir2-deficient cells provided aggregate distribution was solely dependent on random walk. Finally, the distribution and average number of the Htt103Q aggregates observed was similar in wild type and Sir2-deficient cells (Figure 2E) as was the number of heat-induced, Hsp104-associated aggregates (Figure S1). Thus, changes in geometrical parameters, generation time, or aggregate abundance did not explain increased inheritance of aggregates in *sir2*Δ daughter cells.

The passive aggregate diffusion model predicts that cells displaying a reduced growth rate will suffer from a generally increased daughter-cell inheritance of aggregates since the aggregates are allowed a longer time to randomly find their way into, and equilibrate with, the daughter cell. Therefore, we investigated to what extent Htt103Q aggregate inheritance could be enhanced in wild type cells when the generation time was slowed-down after aggregate formation by different concentrations of the protein synthesis inhibitor cyclohexamide. It has been shown that exponential cultures treated with low concentration of cycloheximide do not display arrest in any specific cell cycle stage but instead grow at a slowed exponential fashion with a prolonged cell cycle [26]. Since septum formation occurs only after the completion of mitotic events [27] the bud neck should remain open for a prolonged time upon exposure to low concentrations of cycloheximide. The Htt103Q-GFP reporter is a useful model protein for this experiment (see Figure 2F for the experimental rationale) because Htt103Q aggregates are stable (not cleared) during long periods of time (Figure 1E) and aggregate formation does not involve changes in temperatures, which would affect diffusion rates. The segregation analysis demonstrated that prolonging the generation time more than two-fold did not result in an increased inheritance of Htt103Q aggregates (Figure 2G), suggesting that the establishment of aggregate asymmetry cannot rely on slow and random diffusion alone.

### The genetic interaction network of *SIR2* includes discrete essential genes with a role in actin/tubulin-dependent functions, ER-Golgi trafficking, and chromatid segregation

To approach the passive diffusion model and factor-dependent models further, we next identified which Sir2-dependent functions are involved in restricting aggregate transfer to daughter cells. Therefore, we supplemented the previously identified genetic interaction network of *SIR2* [14] with essential alleles included in the ordered, temperature-sensitive (ts), mutant library reported by Li et al. [28]. The rational for this approach is based on data suggesting that a failure to segregate protein damage can result in a reduced fitness [14],[29],[30] and it has previously been shown [14] that machineries involved in the partitioning of protein damage could be identified among the genes interacting (as synthetic sick or lethal) with a *sir2* deletion using synthetic genetic arrays (SGA) analysis [31]–[33]. The protocol for allowing a *sir2*Δ mutant to mate and produce spores in an SGA screen has been reported previously and includes deletion of the *HMR and HML* silent mating type loci in the *SIR2* query strain [14]. The *sir2*Δ × ts-allele crosses were tested for growth at varying temperatures because different ts-mutants in the library display fitness defects under different semi-permissive conditions.

**Figure 3:**
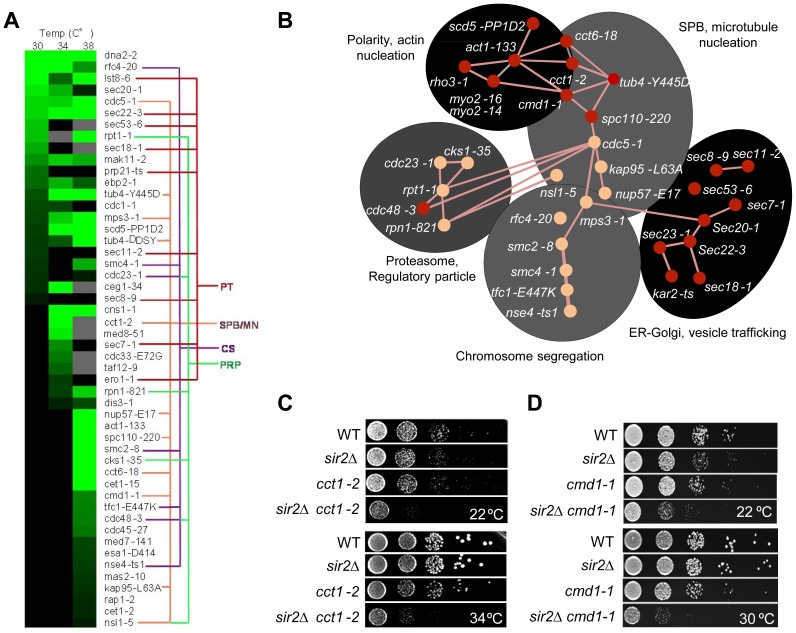
Genetic interactions of *SIR2* with essential genes. **A.** Heat map showing confirmed growth rate changes of *SIR2/*ts essential allele double mutants at 30, 34 and 38°C. The colored lines connecting alleles indicate that the genes belong to the same gene ontology functional group: PT; Protein transport/trafficking, SPB/MN; Spindle pole body and microtubule nucleation, CS; Chromosome segregation, PRP; Proteasome regulatory particle. Green color levels correspond to significant growth rate changes; see Supplemental table S2 for detailed values. Gray indicates no data, black indicates no interaction observed. Data are represented as mean of triplicates. Individual comparisons are made by two-tailed t-test. **B.** Network analysis of the *SIR2* essential interactors. Functional groups are shown as shaded areas. Pink lines indicate published physical interactions and red nodes indicate mutants with aggregate retention defects. **C&D.** Drop tests showing genetic, epistatic, interactions between *SIR2* - *CCT1* and *SIR2* - *CMD1*.

We found that 6% of the 787 alleles included in the ts-library displayed statistically significant negative genetic interaction with *SIR2*. As seen in Table S2 and figures 3A&B, *SIR2* displayed negative genetic interactions with genes involved in actin polarity, actin folding, and actin nucleation consistent with previous results [1], [4], [14], [20]. Analysis of functional relationships and known physical interactions identified 4 additional, previously unknown, functional groups of the *SIR2* interaction network: 1. ‘SPB, microtubule nucleation’, 2. ‘ER-Golgi trafficking/function’, 3. ‘chromosome/sister chromatid segregation’, and 4. ‘proteasome regulatory particle’ (Figure 3A&B).

A *sir2*Δ mutant contains a higher ratio of unfolded/folded actin monomers than wild type cells and the chaperonin CCT isolated from *sir2*Δ cells displays a reduced rate of actin folding [14]. Consistently, the *cct1-2* allele, similar to the *cct6-18* allele [14], was found here to cause severe synthetic sickness in combination with *sir2*Δ (Figure 3C). The CCT chaperonin is also providing the microtubule cytoskeletal system with folded tubulin, which could explain why *tub* mutants are also synthetic sick in combination with *sir2*Δ and why genes of the ‘SPB, microtubule nucleation’ and ‘chromosome/sister chromatid segregation’ functional groups interacts negatively with *sir2*Δ. The *SIR2* interactors of these groups are functionally related and interconnected also by physical interactions between Cdc5, Mps3 and Smc2 (Figure 3B). Mps3 and Cdc5 are required for SPB duplication and separation, respectively, and Mps3 interacts physically with Smc2 of the Smc2/4 condensin complex. Both *smc2* and *smc4* mutants displayed synthetic sickness in combination with *sir2*Δ (Table S2; Figure 3A&B), which is interesting as the cohesin subunit Smc3 displays elevated levels of acetylation in a *sir2*Δ mutant following α-factor treatment [34].

Like CCT, *CMD1*, encoding calmodulin, is required for proper function of both the actin and microtubule cytoskeletons [35]. Consistently, *cmd1-1* mutant cells were severely impaired for growth when combined with *sir2*Δ (Figure 3D).

Essential genetic *SIR2* interactors also included a relative large number of *SEC* genes involved in ER/Golgi functionality and trafficking (Figure 3A&B); specifically, *sec18/20/22* involved in retrograde transport between the ER and Golgi, *sec7*, required for intra-Golgi and ER-to-Golgi transport, *sec53* required for folding and glycosylation of proteins in the ER lumen, and *sec11* needed for targeting proteins to the ER. In line with Sir2 buffering against defects in ER functions, the *cdc48-3* allele encoding a temperature sensitive AAA+ chaperone, which facilitates extraction of ubiquitylated misfolded proteins from the ER, also displayed negative genetic interaction with *sir2*Δ (Table S2; Figure 3A&B). The ‘ER-Golgi trafficking/quality control’ group of genes is more distantly connected functionally to the *CCT/CMD1* groups with respect to genetic interactions [33], [36] suggesting that this group of genes display genetic interaction with *SIR2* for other reasons than defects in CCT and actin/microtubule functionality.

### Asymmetrical inheritance of heat-induced aggregates requires actin-Myo2-calmodulin and ER-Golgi trafficking/functions

**Figure 4:**
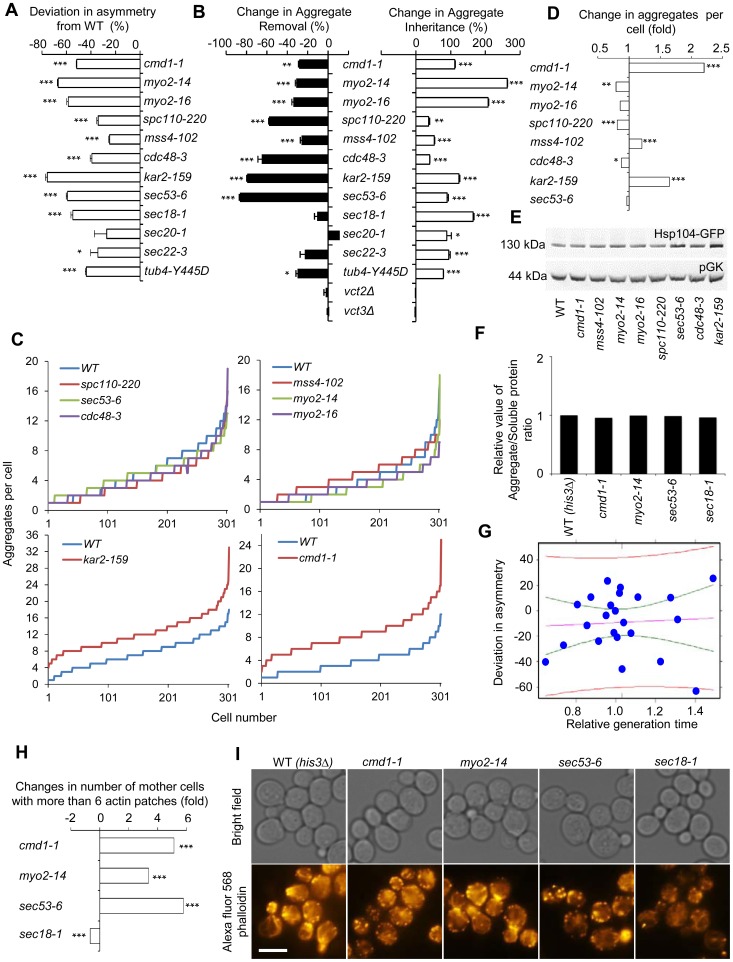
Actin/calmodulin/tubulin, phosphatidylinositol (4,5)-bisphosphate, and ER-Golgi trafficking, are required for asymmetrical inheritance of protein aggregates. A. Temperature sensitive alleles of *SIR2* essential interactors that display a reduced ability to establish aggregate asymmetry during cytokinesis. Negative values indicate a reduction in the percentage of daughter cells generated without aggregates compared to wild type. B. Alterations in aggregate inheritance and removal in the mutants displaying defects in establishing aggregate asymmetry. Data are plotted as the mutants' deviation from the wild type in aggregate inheritance (open bars; 100% increase means a two-fold increase in the aggregate inheritance values compared to wild type strains) and removal (black bars; 100% decrease means a two-fold decrease in the inheritance values compared to wild type strains). C&D. Distribution of aggregate numbers in populations of wild type and mutant cells displaying defects in aggregate inheritance. E. Hsp104 levels detected by Western blot in mutants displaying defects in aggregate inheritance. The western blot probed with anti-Hsp104 antibody is shown in the upper panel and the loading control probed with anti-pGK antibody is shown in the lower panel. F. Relative levels of aggregated/total proteins after the heat shock in different ts-allele strains. G. Analysis of Hsp104-GFP aggregate inheritance as a function of the generation time obtained during the aggregate segregation tests of the ts-mutants. Vastly different degrees of inheritance were recorded within the confidence interval, and the best linear fit shows that no statistically significant correlation trend can be observed (R^2^ = −0.04346 and P = 0.7752). The data (blue dots), the fit line (purple), the confidence interval lines (green), and the predicted interval lines (red) for a linear regression analysis are displayed. H. Polarity defects in ts-mutants displaying aberrant aggregate segregation. Three of four tested ts-mutants, *cmd1-1*, *myo2-14* and *sec53-6*, displayed polarity defects as seen by an increase number of mother cells with more than 6 actin patches. Fold changes are calculated from 200–300 cells. The statistical significance of observed differences was determined with the two-tailed U-test. I. Pictures of ts-mutants tested for polarity defect. Upper panel shows bright field images and lower panel shows polarity phenotypes visualized by phalloidin staining. Scale bar  = 5 µm. Asymmetry, aggregate inheritance and removal and change in aggregate per cell data are presented as mean + s.d. of triplicate samples. Statistically significant differences from wild types are determined by unpaired two-tailed *t*-test. Asterisks denote significant differences between samples: *P,0.05; **P,0.01; ***P,0.001.

By crossing the *HSP104-GFP* fusion into the essential ts-mutant library using synthetic genetic array technology, we next tested whether any of the functional groups of the essential *SIR2* genetic interaction network displayed aberrant aggregate inheritance of heat-induced Hsp104-associate aggregates and then followed up by testing if asymmetrical Htt103Q inheritance required the same factors. Among all the essential alleles interacting with *SIR2*, about 40% caused a defect in establishing Hsp104-aggregate asymmetry. One of the most severely affected mutants, *cmd1-1*, encoding calmodulin, belong to the group of genes involved in actin cable organization and function (Figure 4A). In addition, defects in the organization of both tubulin *(tub4-Y445D)* and the SPB *(spc110-220)* affected asymmetry (Figure 4A), suggesting that the machineries required for nuclei segregation are also required for establishing aggregate asymmetry. This is consistent with data demonstrating that aberrant nuclei segregation can lead to daughter-cell inheritance of protein inclusions, especially JUNQ [6]. With the exception of *cdc48-3*, mutants of the ‘proteasome regulatory particle’ and ‘chromosome/sister chromatid segregation’ groups did not display aberrant aggregate asymmetry, whereas all alleles in the ‘ER/Golgi trafficking/function’ group did (Figure 4A).

Calmodulin regulates many processes apart from actin cable organization, including vacuole inheritance, endocytosis, microautophagy, and organization and formation of the SBP. Therefore, we next tested if any or all of these processes/components are either required for preventing the inheritance of aggregates (retention in mother cells), clearance of aggregates (in daughter cells), or both using the ConA protocol (see Figure 1). Mutations in *CMD1* have been reported to cause actin cytoskeletal defects by reducing the levels of the signaling molecule phosphatidylinositol (4, 5)-bisphosphate [37]. We found that the *sir2*Δ interactor *mss4-102*, a mutant allele of the phosphatidylinositol (4, 5)-bisphosphate kinase, increased aggregate inheritance and decreased aggregate removal in daughter cells (Figure 4B). In addition, Cmd1 is required for polarized growth and inheritance of the vacuole by daughter cells through its interaction with the type V myosin motor protein Myo2 [38], [39], and cells harboring the *myo2-14* or *myo2-16* alleles, like *cmd1-1* cells, displayed severe defects in both aggregate inheritance and removal (Figure 4B). Likewise, Spc110, which requires Cmd1 for its proper localization to the SPB [35], [40], and tubulin (*tub4-Y445D*) were required for both asymmetrical inheritance and removal of aggregates (Figure 4B). In contrast, deficiencies in Cmd1-dependent microautophagy, which is mediated by Vtc2 and Vtc3 [41], were not affecting aggregate asymmetry (Figure 4B).

Among the calmodulin-independent genes of the *SIR2* interaction network, all involved in ER/Golgi trafficking/functionality and the UPR/ERAD, displayed deficiencies in establishing aggregate asymmetry (Figure 4A) and by testing some selected alleles in this group including *sec53-6*, *sec20-1, sec22-1, sec18-1*, *kar2-ts*, and *cdc48-3* using the conA protocol we found that all these genes were required for preventing aggregate inheritance in daughter cells (Figure 4B).

The mutations identified causing an increased daughter-cell inheritance of protein aggregates could be doing so by affecting aggregate numbers if aggregate partitioning is predominantly due to random diffusion. Therefore, we quantified aggregates in the mutants of the functional groups found to be required for asymmetrical inheritance. This analysis demonstrated that the absence of most genes identified here as being required for aggregate asymmetry, did not significantly increase aggregate numbers (Figure 4C&D, Figure S2). However, there are some intriguing exceptions; reduced activity of Cmd1 and the ER chaperone Kar2 caused a marked increase in the average number of aggregates per cell indicating that these proteins are required for inclusion body formation (Figure 4C&D, Figure S2). Nevertheless, alterations in aggregate inheritance in the majority of the mutants identified are uncoupled from changes in aggregate numbers. Defects in aggregate partitioning could also be due to diminished levels of Hsp104 [4], [5]. However, for the mutants tested herein, the defects in inheritance was not accompanied by reduced Hsp104 levels (Figure 4E), or elevated total levels of insoluble proteins, which were separated from soluble proteins by ultracentrifugation (Figure 4F).

To test to what extent alterations in generation times might contribute to changes in aggregate inheritance, we recorded daughter cell inheritance for the ts-mutants analyzed as a function of the generation time obtained during the aggregate segregation analysis. The mutants and temperatures analyzed generated generation times within a 1.5 fold difference from the wild type cells. The data was subjected to linear regression analysis together with confidence and prediction interval determinations to quantify the contribution of generation times on inheritance. A number of important observations can be made from this analysis. First, within the confidence interval (i.e. the interval displaying little difference in generation times) vastly different degrees of inheritance were recorded (Figure 4G), demonstrating that the effects on inheritance must be governed by other means than alterations in the generation time within this group of mutants. Second, in contrast to the predictions of the passive diffusion model, the best linear fit shows a weak trend towards a decreased inheritance with increased generation times but the adjusted R-squared value and p-value of −0.04346 and 0.7752, respectively, demonstrate that this trend is not statistically significant.

To test if the segregation defect seen in *sec* mutants could be linked to aberrancies in actin cytoskeleton organization, we analyzed actin polarity as described in [42], and found that *sec53-5*, like *cmd1 and myo2* mutants, displayed a markedly aberrant actin polarity (increase number of cells with more than 6 actin patches) whereas the *sec18-1* mutant showed a decreased number of patches (Figure 4H&I). Thus, it is possible that some SEC and ER-associated mutants fail to segregate aggregates asymmetrically due to polarity defects.

### Htt103Q forms Cmd1/Myo2/actin-associated foci requiring Cmd1, Myo2, and *SEC* genes for their mother cell-biased segregation

**Figure 5:**
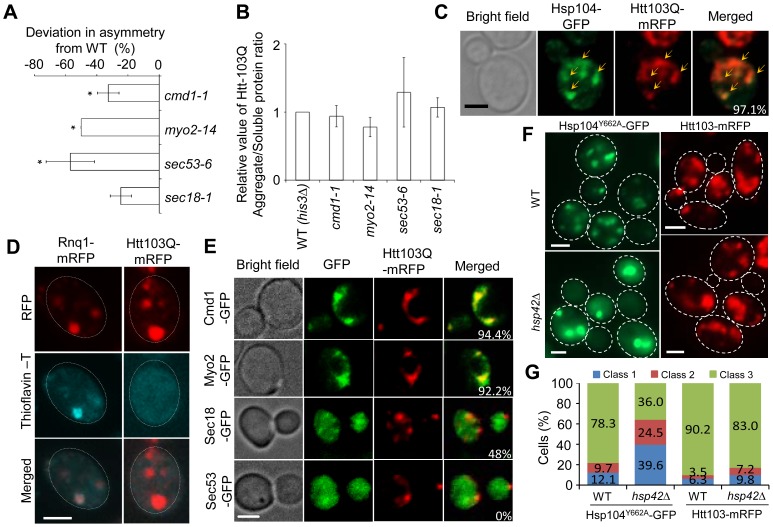
Htt103Q foci associate with Cmd1/Myo2/actin and require Cmd1, Myo2, and SEC genes for their mother cell-biased segregation. A. *SIR2* essential interactors *cmd1-1, myo2-14, sec53-6* and *sec18-1* display a reduced ability to establish mother-biased segregation of Htt-103Q aggregates. Negative values indicate a reduction in the percentage of daughter cells without aggregates compared to the wild type. Asymmetry data are presented as mean + s.d. of duplicate samples. Statistically significant differences from wild types are determined by unpaired two-tailed t-test. Asterisks denote significant differences between samples: *P,0.05. B. Relative amount of aggregated/insoluble Htt103Q proteins in the different ts-allele strains compared to that of wild type cells after induction of Htt103Q with 2% galactose. Statistically significant differences from wild types were determined by unpaired two-tailed *t*-test. C. Representative images showing co-localization (yellow arrows) of Hsp104-GFP (green) and Htt103Q-mRFP (red). Cells were heat-shocked at 42°C and allowed to recover for 30 minutes at 30°C. The co-localization of these two aggregates can be observed in about 97.1% cells displaying both type of aggregates D. Amyloid staining of cells carrying Htt103Q aggregates (right column) using Thioflavin–T. Images of cells expressing Rnq1-mRFP were used as a positive control for amyloidogenic protein aggregates and are shown in the left column. E. Co-localization of Htt103Q-mRFP aggregates with specific proteins found to be required for mother-biased segregation as indicated. The two upper panels show that Cmd1-GFP and Myo2-GFP structures overlap and co-localize with Htt103Q-mRFP aggregates in cells showing both mRFP tagged aggregates and GFP tagged structures (94.4% and 92.2% cells showed Htt103Q-mRFP co-localization with Cmd1 and Myo2 structures, respectively). The bottom two panels indicate that less cells showed co-localization between Htt103Q-mRFP and Sec18-GFP (48%) and no co-localization between Sec53-GFP and Htt103Q-mRFP aggregates. F. Heat-denatured proteins associated with Hsp104^Y662A^-GFP (green) but not the Huntington protein Htt103Q-mRFP (red) required Hsp42 for foci formation. Upper panels: wild type cells, lower panels: *hsp42*Δ mutants. G. Quantification of Hsp104^Y662A^ and Htt103Q aggregate morphology changes in WT and *hsp42*Δ cells. More than 100 cells from Z-stack images showing aggregates were quantified. Cells were divided into 3 classes (Class 1, cells with 1 aggregate; Cclass2, cells with 2 aggregates; Class 3, cells with 3 or more aggregates). Scale bar  = 5 µm.

We next tested selected alleles that markedly reduced mother cell-biased segregation of heat-induced Hsp104-associated aggregates for their effect on asymmetrical segregation of Htt103Q. We found that both Cmd1 and Myo2, as well as the *SEC* genes (*SEC18* and *SEC53*) were required for asymmetrical segregation of Htt103Q (Figure 5A). As for heat induced Hsp104-associated aggregates, this defect was not due to elevated levels of unfolded and insoluble Htt103Q in these cells (Figure 5B). The requisite of the same factors for asymmetrical segregation of both heat-induced Hsp104-associated aggregates and Htt103Q is somewhat unexpected as the former is sequestered into distinct, inclusion bodies (IBs), IPOD and JUNQ, upon heat stress [7], whereas Htt103Q forms multiple small aggregates throughout the cytoplasm [23], [24], [43]. However, before the formation of IPOD/JUNQ, misfolded, Hsp104-associated, proteins assemble into small stress foci ([6]; also called Q-bodies [30] or peripheral aggregates [44]), reminiscent of the smaller Htt103Q aggregates. We therefore tested if Hsp104-GFP co-localized with Htt103Q immediately after heat stress and found this to be the case; co-localization can be observed in about 97.1% cells displaying both Htt103 and Hsp104 aggregates (Figure 5C), indicating that Htt103Q may be sequestered at terminally stable stress foci-like structures. It has been suggested that amyloids and heat-denatured proteins are sequestered to spatially different quality control sites [7]. Therefore, we tested whether Htt103Q formed amyloids using Thioflavin-T staining but found no evidence for this whereas the positive control, Rnq1-mRFP aggregates readily stained with Thioflavin-T (Figure 5D).

Next, we analyzed if Htt103Q foci co-localized with Cmd1 and/or Myo2, which could explain, in a direct physical manner, why retention in the mother cell relies on these factors. In cells where Cmd1 or Myo2 were enriched in visible structures, 94.4% and 92.2% showed co-localization between Htt103Q and such Cmd1 or Myo2 structures, respectively (Figure 5E). In addition, the ATPase-deficient Hsp104, Hsp104^Y662A^, which has previously been shown to be ‘locked’ in a stress foci stage [6], similarly co-localized with Cmd1 (in 74.4% of cells) and Myo2 (in 56.6% of cells) enriched structures (Figure S3). We found less co-localization between Htt103Q foci and Sec18 (about 48% of cell showing both Sec18 structures and Htt103Q foci) whereas no clear co-localization could be observed between Htt103Q foci and Sec53 (displaying a diffuse signal) (Figure 5E). In contrast, in cells with heat induced Hsp104^Y662A^ foci a clear co-localization can be observed between Hsp104^Y662A^ and Sec53 (82.7%; Figure S3). Some of the Htt103Q and Hsp104^Y662A^ foci appeared to reside in the vicinity of the ER, as detected by Rtn1-GFP co-staining (Figure S4). Previous studies have shown that the small heat shock protein Hsp42 affects sequestration of misfolded proteins; specifically, in the absence of Hsp42 misfolded proteins are predominantly directed to the juxtanuclear JUNQ deposition site instead of peripheral, nucleus-distant, aggregation sites [8], [44]. Importantly, we found that the absence of Hsp42 redirected Hsp104^Y662A^ to inclusions (cells with 1 or 2 aggregates; i.e. class 1 and 2 cells) rather than peripheral aggregates/stress foci (3 or more aggregates; class 3 cells) whereas formation of Htt103Q foci was unaltered (Figure 5F,G). Co-staining with DAPI demonstrated that the number of cells with a single juxtanuclear-localized aggregate were increased in the *hsp42*Δ mutant (Figure S5). These data indicate that while Hsp104^Y662A^ and Htt103Q are directed to overlapping, Cmd1/Myo2-associated, foci, the routes/factors employed for sequestering heat-induced stress foci and Htt103Q to such sites may be different.

**Figure 6:**
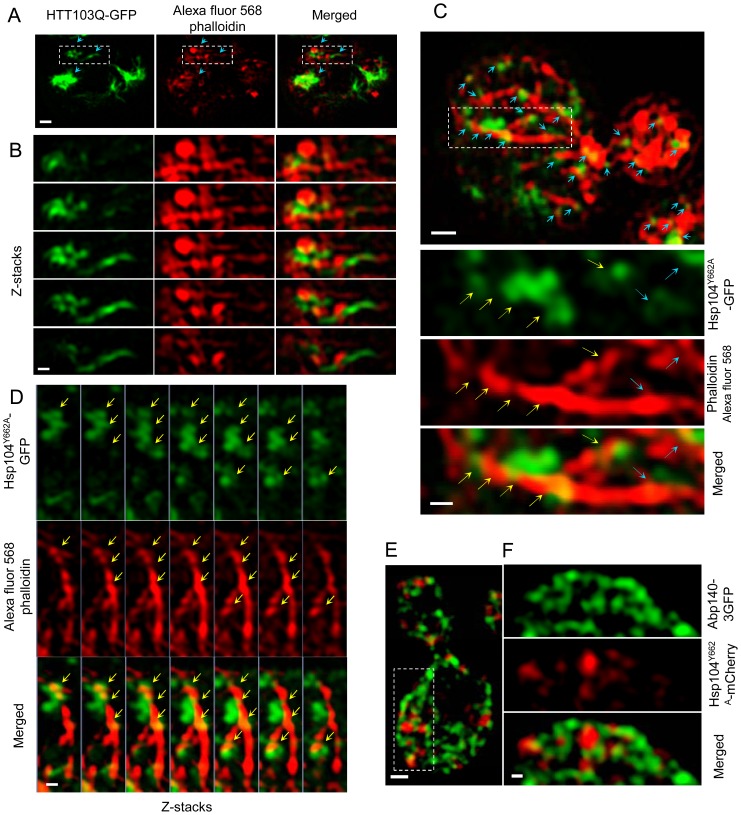
Super-resolution three-dimensional structured illumination microscopy (3D-SIM) revealing association of Htt103Q and Hsp104-linked stress foci with the actin cytoskeleton. A&B. 3D-SIM images show that Htt103Q-GFP (green channel) aggregates are associated with the actin cytoskeleton (red channel: Alexa fluor 568 phalloidin). Blue arrows indicate regions where aggregates are in close proximity to the actin cytoskeleton. C. 3D-SIM analysis of Hsp104^Y662A^-GFP stress foci after a heat shock. Blue arrows indicate that Hsp104^Y662A^-GFP-associated foci (green channel) are lining up along actin cables (red channel: Alexa fluor 568 phalloidin) and are at some places wrapping around the cable (yellow arrows). D. Z-stack series of Hsp104Y662A-GFP, Alexa fluor 568 phalloidin and Merged channels. Z stacks were collected using a 100 nm optical section thickness. Yellow arrows indicate locations in which aggregates (green) are in close proximity to, and/or wrapping around, actin cables (red). E&F. 3D-SIM microscopy shows that protein aggregates (Hsp104^Y662A^-mCherry, red) are associated with the Abp140-3GFP (green) actin cable-binding protein. Scale bars in whole cell images  = 0.5 µm, scale bars in zoomed images  = 0.2 µm.

Cmd1 and Myo2 are intimately associated with the actin cytoskeleton and protein aggregates and prions have been found previously to reside in areas rich in actin-enriched structures using proximity ligation assays and co-localization fluorescence microscopy [14], [18], [19], [43], [45]. However, one major drawback with conventional fluorescence microscopy is that the *x*–*y* axial resolution is limited to about 250 nm and the z axial resolution to about 500 nm [46], [47]. Therefore, to more precisely analyze the spatial relationship between protein aggregates/stress foci and the actin cytoskeleton, we performed super-resolution three-dimensional structured illumination microscopy (3D-SIM) [48] to analyze possible aggregate and actin cytoskeleton interactions *in vivo*. With this technique an approximately 8-fold smaller volume can be resolved in comparison to conventional microscopy equating about 100 nm in *x*-*y* and 200 nm in the *z* axial [46], [49]. The 3D-SIM analyses revealed that both Htt103Q and Hsp104^Y662A^ foci line up along actin cables and are in some instances wrapping around the cables (Figure 6A–D, Figure S6 and Movie S1). At this resolution, using multiple Z-stacks, it is clear that the co-localization is not due to actin oligomers residing in the aggregates themselves (Figure 6B&D). Moreover, we found that Hsp104^Y662A^-mCherry stress foci displayed a considerable co-localization with the actin cable-associated protein Abp140-3GFP further supporting an association between stress foci and actin cables (Figure 6E&F).

## Discussion

The development of an *in situ* protocol for detecting oxidatively damaged (carbonylated) proteins in single cells of *S. cerevisiae* led to the discovery that damaged proteins display a mother cell-biased segregation during cytokinesis [1] and it was later shown that such oxidatively damaged proteins coalesce into aggregates upon aging that rely on both Hsp104 [4], [5] and Sir2 [1], [14], [50] for their asymmetrical inheritance. Data accompanying the original discovery showed also that elevating damage in the mother cell by a transient exposure to oxidants rendered asymmetrical inheritance even more pronounced indicating that asymmetry was not entirely due to a passive effect of slow diffusion [1]. The data presented in this work is consistent with this notion: If aggregates would find their way into a daughter cell purely by passive and random movement, increasing the time for completing cytokinesis would enhance aggregate inheritance - we show here that this is not the case. Further, mutants with increased daughter-cell inheritance should, if aggregates diffuse randomly, either display a larger bud-neck diameter, a longer generation time, or increased number of aggregates; none of these traits could be established for *sir2Δ* cells or the other mutants displaying elevated inheritance. Prevention of inheritance of both the misfolded polyQ protein Htt103Q and heat-induced aggregates relies instead on specific cellular processes/components, including the actin-associated proteins Cmd1 and Myo2 and Sec proteins involved in ER to Golgi trafficking and ER homeostasis.

It is possible that a reduction in actin cable abundance affects aggregate diffusion, as an intact actin cytoskeleton in *dictyostelium* appears to slow down the diffusion rates of soluble GFP proteins [51]. However, since both Htt103Q and heat-induced foci co-localized with Cmd1- and Myo2-enriched structures, the retention of aggregates might be linked to a more direct physical interaction between these components and aggregates. Cmd1 and Myo2 are intimately associated with actin cables and super-resolution 3-D SIM microscopy demonstrating that stable foci of both Htt103Q and Hsp104^Y662A^ are associated with the actin cytoskeleton (and the actin-binding protein Abp140) in the nm-scale. These data is further supporting a role of actin cable assembly [1], [4], [44], actin folding [14], and actin polarity [19] in aggregate inheritance control.

Interestingly, the Htt103Q foci co-localized with the Hsp104 stress foci formed early upon a heat shock. Thus, misfolded Htt103Q and heat-denatured proteins appears to be sequestered into the same spatial sites. In further support of this notion, the disaggregase-defective Hsp104^Y662A^-GFP, which upon heat stress forms stable stress foci [6], like Htt103Q, co-localized with Cmd1 and Myo2. However, we found that only Hsp104^Y662A^-associated misfolded proteins, and not Htt103Q, required Hsp42 for foci formation. The absence of Hsp42 has been shown previously to redirect heat-denatured misfolded proteins to the nucleus-proximal JUNQ deposition site at the expense of peripheral aggregation sites at least in the presence of a proteasome inhibitor [8], [44] but we found that Htt103Q foci formation was unaffected by Hsp42 deficiency. Inversely, we found that Cmd1- and Kar2-deficiency reduced the cells' ability to form Hsp104-associated inclusions upon a heat shock; that is, heat-induced aggregates appear ‘locked’ in the stress foci stage in these mutants.

The participation of the Myo2 motor protein and calmodulin in asymmetrical inheritance of aggregates suggests that the role of actin cables and polarity in this process may be linked also to vesicle/organelle trafficking. In support of this notion, the IPOD inclusions are associated with the vacuole [6], [7] and it is conceivable that misfolded proteins reach such deposit sites in an actin cytoskeleton- and vesicle trafficking-dependent way. Indeed, Specht et al., [44] have demonstrated that misfolded proteins fail to form peripheral aggregates when actin cables are depolarized with Latranculin and Kaganovitch et al., [7], using benomyl treatment, demonstrated the requirement also of microtubule in the formation of inclusion bodies. However, it was later shown that the effect of benomyl in inclusion body formation might be microtubule-independent [44]. The effect of abrogated ER/Golgi function on aggregate segregation could also be linked to effects on actin/calmodulin/Myo2-dependent vesicle/vacuole trafficking since the ER/Golgi is involved in lipid modifications of specific proteins, e.g palmitoylation and myristoylation, required for anchoring Myo2 to targets at vesicle membranes [52]. In this scenario, Myo2 might act as a tethering factor required for misfolded/aggregated proteins to become linked to actin cables and/or deposition sites on the surface of the vacuole since misfolded proteins have been demonstrated to associate with membrane vesicles [17], [43]. In addition, a recent report shows that misfolded Ubc9^ts^ proteins form puncta called Q-bodies that are associated with ER [30]. However, it should be noted that Ubc9^ts^-Q-bodies move in an actin-cable-independent (but energy-dependent) manner suggesting that these structures are not themselves associated with actin. The apparent difference with respect to actin-association of Ubc9^ts^ Q-bodies and Htt103Q foci is interesting and may suggest that different misfolded proteins are sequestered to different spatial locations. Another possible reason for the different results is the use of different protocols; whereas Htt103Q readily aggregate upon its production Ubc9^ts^ aggregation is triggered by elevating the temperature, a protocol that disrupts actin cables. Also, while Ubc9^ts^ Q-bodies move in an actin cable-independent manner [30], it is not clear if their subsequent progression to IPOD/JUNQ inclusion sites require functional actin cables or not since the dynamics, morphology and inheritance of cortical ER (which the Ubc9^ts^ Q-bodies associate with) have been linked to actin cytoskeleton components [53]–[55]. Elucidating the exact cytological, biochemical, and genetic nature of stress foci, Q-bodies, peripheral aggregates, and IPOD/JUNQ inclusions and their relevance for different aggregate reporters appears an important task for future research.

It has recently been shown that aggregate accumulation during replicative aging of mother cells follow a delineated path; virgin and young cells display no protein aggregates, middle-aged mother cells harbor one to two protein inclusions, first JUNQ then also IPODs, while old cells display, in addition to JUNQ and IPODs, multiple peripheral aggregates resembling stress foci [56]. It will be interesting to learn to what extent these foci are connected to Cmd1/Myo2 and the actin cytoskeleton and if such associations are actually a cause of aging. We envision that the tethering of multiple aggregates will disturb actin cable-dependent trafficking processes and eventually cause a complete collapse in the physical integrity of the actin cytoskeleton. In addition, the new and previously unknown genetic interactions between *SIR2* and essential genes recorded herein points to additional Sir2-related functions of potential relevance for life span control. Specifically, since Sir2 buffers against deficiencies in microtubule/spindle pole body and chromosome/sister chromatid segregation functions, it is tempting to speculate that the diminishing level/activity of Sir2 observed in aging cells [57] leads to problems also in performing proper chromosome/nuclei segregation. Further studies appear warranted to elucidate how this sirtuin is mechanistically buffering against defects in these essential functions and how they might relate to sirtuins acting as gerontogenes.

## Materials and Methods

### Yeast strains and growth conditions

Yeast strains used in this study are listed in Supplemental Table S1. The Yeast conditional temperature-sensitive (ts) collection of essential genes for the SGA analysis was a gift from Prof. Charles Boone. Yeast cells were grown in YPD or synthetic drop-out media with antibiotics added as indicated.

### SIR2 Synthetic Genetic Array (SGA) analysis using the conditional temperature-sensitive (ts) collection of essential genes

The *SIR2* (ts) SGA analysis was performed in duplicates as described [28]. The screen was run in the 1536-spot format using a SINGER ROTOR HDA Robot (Singer Instrument Co. Ltd.). Hits with the highest statistical probability to be true interactions were confirmed by microcultivation experiments in triplicate at 30, 34, and 38°C using the Bioscreen C system (Labsystems Oy, Helsinki, Finland). The optical density was measured every 30 minutes for 72 hours. The LSC (Logarithmic Strain Coefficient) values of growth rates were calculated and scored as described [28]. The heat map was made using TreeView [58] and Ospery 1.2.0 [59] was used for *SIR2* essential gene network analysis. The physical interactions between SGA hits were obtained based on the BioGRID interaction database [60].

### Fluorescence microscopy

A Zeiss Axiovert 200 M fluorescence microscope was used to obtain images using GFP, Cy3 and DAPI channels. The ImageJ plugin “Iterative deconvolve 3-D” was used for all deconvolution images.

### HttQ103-GFP expression and aggregation retention efficiency assay

Cells containing the pYES2-HttQ103-GFP plasmid were grown at 30°C to exponential phase (OD_600_ about 0.5) in YNB-URA 2% raffinose. Htt103Q-GFP expression was induced by adding galactose to a final concentration of 2%. After 4 hours at 30°C cells were washed and resuspended in 1.5 ml Buffer P (10 mM NaH_2_PO_4_, 150 mM NaCl, 2% galactose; pH 7.2). All cells present during the expression of Htt103Q-GFP were marked by staining the cell wall components α-mannopyranosyl and α-glucopyranosyl with 0.2 mg/ml concanavalin A Alexa Fluor 647 (Invitrogen) for 30 minutes at room temperature. The cells were then washed in Buffer P, resuspended in YNB-URA with 2% glucose, which will switch off the Htt103Q-GFP expression, and grown at 30°C for one budding event. This makes it possible to distinguish between concanavalin A stained daughter cells present during Htt103Q-GFP expression and daughter cells produced subsequent to Htt103Q-GFP expression. Cells were fixed in 3.7% formaldehyde and segregation of aggregates was analyzed using fluorescence microscopy. The segregation assay of ts-alleles were performed in the same way but without concanvalin A staining. Cells were grown at 22°C to exponential phase, followed by induction and budding at different temperatures (26°C for *sec18-1* and *sec53-6*, 28°C for *cmd1-1* and 32°C for *myo2-14*).

### Removal and retention assay of heat induced aggregate using concanavalin A staining

The retention efficiency assay was performed as described [14] with concanavalin A staining (as described above) before the heat shock treatment. Aggregate retention and removal was distuinguished upon image analysis. Retention is determined as the percentage of aggregate-containing buds of the total number of buds generated from an aggregate-containing mother cell after the heat shock treatment (buds free of conA); Removal efficiency is determined as the perecentage of aggregate-free buds of the total number of buds already existing before heatshock; i.e. stained with conA. More than 300 budding events were quantified for each strains.

### Statistical analysis of aggregate segregation as a function of the generation time

All statistical calculations were done in R-3.0.0 (www.r-project.org). Regression analysis was performed and showed that there is no statistically significant difference at p<0.05 to test that whether there is a correlation between ‘relative generation time’ and ‘deviation in asymmetry’.

### mHtt103Q stability measurement

The *in vivo* expression of mHtt103Q-GFP protein was induced by galactose addition to yeast cells in mid-exponential phase (OD_600_ = 0.5) grown in media with 2% raffinose. 10 µg/ml of cycloheximide (CHX) was added into the culture after 3 hours of induction to stop translation. Cells were continued to be cultured at 30°C with shaking. Aliquots were then taken and fixed at 0, 1, 2, 3, 4 and 12 hours after the addition of CHX. GFP signal intensity for each sample was quantified by flow cytometry (FACS Aria, BD equipment). 10000 events were counted for each sample. A cut-off value was picked based on an un-induced control. Stability is measured as mean signal intensity from Htt103Q-GFP aggregates as a function of time after inhibition of protein synthesis by adding CHX.

### Aggregate inheritance after increasing generation times by cycloheximide

Cells containing the pYES2-HttQ103-GFP plasmid were grown at 30°C until OD_600_ reached approximately 0.5 in SC-URA +2% raffinose. The expression of Htt103Q-GFP was induced by adding 2% galactose for 4 hours at 30°C. Cells were then stained with ConA as described above. The cell density was adjusted to OD_600_ = 0.5. The cells were divided into 3 groups and recovered at 30°C for different times until the cells grow to the same optical density value as the untreated control with different concentrations of cycloheximide. Further expression of HttQ103-GFP was inhibited by adding 2% glucose to the medium during bud formation. The cycloheximide treatment was performed as follows: In the untreated group, no cycloheximide was added during the budding period and the cells were recovered for 4 hours at 30°C to let the cells generate new buds and grow to a OD_600_ reaching 0.8. In the two cycloheximide treatment groups, 0.05 or 0.1 mg/ml cycloheximide were added to the cell cultures and grown at 30°C until OD_600_ reaches the same value (0.8) as in the untreated control group. Then cells were fixed in 3.7% formaldehyde and budding events with newly generated buds were analysed for aggregate inheritance using a Zeiss fluorescence microscope.

### Western blot

20 mL OD600 = 0.7 Yeast cells were collected after heat shock and recovery then resuspended in 800 µl 0.1 M NaOH for 5 minutes (room temperature) and then pellet. The cells were boiled for 3 minutes in 125 µl lysis buffer (50 mM Tris, pH 7.4, 5 mM EDTA, 5 Mm NEM, 1% SDS) with protease inhibitor (Roche, 11697498001). The supernatant was mixed with equal amount laemmli buffer and heated at 95°C for 3 minutes. Denatured proteins were loaded onto NuPAGE Novex 10% Bis-Tris Gels (Invitrogen, NP0315BOX) and transferred to PVDF membranes to perform western blotting. Antibody to GFP (Roche, 11814460001) and pGK (Invitrogen, 459250, as loading control) were used in this study. After western blotting, the membranes were scanned by Odyssey Infrared Imaging System and quantified by Odyssey 2.1 software. Fold ratios were calculated based on three biological repeat experiments.

### Solubility assay

Solubility assays were carried out as described in [61]. Same volume of protein solution of each sample was loaded on precasted SDS-PAGE gels (Life Technologies). For testing heat-induced aggregates, the gel was then stained by Coomassie Brilliant Blue and scanned with a GS-800 Calibrated Densitometer (BioRad). For Htt-103Q-GFP expressing cells, the Htt-103Q-GFP protein was detected by Western blotting with mouse anti-GFP monoclonal antibody (Roche). The scanned gels and Western membranes were then quantified by ImageJ software. Relative ratio of soluble and aggregated protein for each tested strain were then calculated and plotted.

### Thioflavin T staining

Thioflavin T staining of amyloid was performed according to a protocol from [62] with minor changes. Cells were fixed in 50 mM KPO_4_ (pH 6.5), 1 mM MgCl_2_, 4% formaldehyde for 10 minutes and then washed three times with PM buffer [0.1 M KPO_4_ (pH 7.5), 1 mM MgCl_2_] and resuspended in PMST [0.1 M KPO_4_ (pH 7.5), 1 mM MgCl_2_, 1 M Sorbitol, 0.1% Tween 20]. Cells were treated with 0.125 mg/ml Zymolase at room temperature for 15 minutes in the presence of 0.6% beta-mercaptoethanol. Spheroplasted cells were washed once and then resuspended in PMST and stained with 0.001% Thioflavin T for 20 minutes at room temperature. After five times of washing with PMST, the cells were observed under a Zeiss Observer Z1 microscope at CFP channel for the staining.

### Hsp104-Y662A-mCherry aggregate induction

Mid-log phase cells (OD = 0.5) were incubated at 42°C for 10 minutes and then 30°C for 30 minutes. These cells were then fixed with 3.7% formaldehyde and washed twice with PBS (pH 7.4) and stored for microscopy.

### Quantification of co-localization and aggregate morphology

At least 100 cells with both visible structures (formed by GFP-tagged proteins) and red aggregates (formed by Hsp104-Y662A-mCherry or Htt103Q-mRFP) were analyzed. Among them, cells with any overlapping green and red signals were considered as cells with co-localization, the percentage of which were then calculated. Z-stack images were used for aggregate morphology quantification and more than 100 cells showing aggregates were quantified. Cells were divided into 3 classes based on the number of aggregates in the cell (Class 1, cells with 1 aggregate; Class2, cells with 2 aggregates; Class 3, cells with 3 or more aggregates). Aggregates were counted throughout all Z-stacks.

### Actin depolarization analysis

Actin depolarization was quantified according to Ho *et al*. [42]. All budding events that have a small or medium bud were counted for number of actin patches in the mother cells. Mother cells bearing more than 6 actin patches were counted and the percentage of them over all mother cells were calculated and plotted.

### Super-resolution three-dimensional structured illumination microscopy (3D-SIM)

Cells carrying Hsp104^Y662A^-mCherry were incubated at 42°C for 10 minutes and then 30°C for 10–20 minutes. And cells with Htt103Q-GFP was induced by adding 2% galactose for 4 hours at 30°C. Then cells were fixed with 3.7% formaldehyde and washed twice with PBS (pH 7.4). Actin cytoskeleton was stained using Alexa Fluor 568 Phalloidin (Ivitrogen, A12380) as described in the manual. Cells expressing both Abp140-3GFP and Hsp104^Y662A^-mCherry were heat treated as above. For super-resolution three-dimensional structured illumination microscopy, the ELYRA PS.1 LSM780 setup from Zeiss (Carl Zeiss, Jena Germany) was used. 3D-SIM images of the protein aggregates (Hsp104^Y662A^-GFP) and actin cytoskeleton (Alexa fluor 568 phalloidinor Abp140-3GFP) were taken with 100×/1.46 Plan-Apochromat oil-immersion objective with excitation light wavelengths of 488 nm and 561 nm. *Z*-stacks with an interval of 100 nm were used to scan the whole yeast in 3D-SIM. For acquisition and super-resolution processing and calculation as well as for 3D reconstruction, the Zen2011 software (Carl Zeiss, Jena Germany) was used. The ELYRA System was corrected for chromatic aberration in *x*-, *y*-, and *z*-directions using multicolor beads, and all obtained images were examined and aligned accordingly.

## Supporting Information

Figure S1 Distribution in the number of protein aggregates (Hsp104-GFP) per cell in wild type (orange) and *sir2*Δ (red) populations. The average number of aggregates per cell was 11.88 and 11.4 in the wild type and *sir2*Δ mutant, respectively. Average values are calculated from 300–400 cells. The statistical significance of observed differences was determined with the two-tailed U-test. P-values are indicated in the figure. (Related to Figure 2E).
(TIF)

Figure S2 Representative images showing Hsp104-GFP aggregates (bottom panel) in the wild type (*his3*Δ) and ts mutant cells tested. **A&B.** Mutants were tested in 2 separate sets of experiments. Bright field images are showed in the upper panel. Scale bar  = 5 µm. (Related to Figure 4).
(TIF)

Figure S3 Hsp104^Y662A^-mCherry aggregates co-localize with some proteins encoded by essential genes required for asymmetrical aggregate segregation. As shown, some Cmd1-GFP (74.4%, tope panel) or Myo2-GFP (56.6%, second panel from top) structures co-localize with Hsp104^Y662A^-mCherry aggregates. The co-localization of Sec18-GFP and Hsp104^Y662A^-mCherry aggregates can be observed in only about 16.8% of cells (third panel). Sec53-GFP structures overlaps with some Hsp104^Y662A^-mCherry aggregates (82.7%). Scale bar  = 5 µm. (Related to Figure 5).
(TIF)

Figure S4 Cortical and cytoplasmic Rtn1-GFP signals show partial overlap with Hsp104Y662A-mCherry and Htt103Q-mRFP aggregates. Scale bar  = 5 µm. (Related to Figure 5).
(TIF)

Figure S5 The *hsp42*Δ mutant displays increased number of cells with a single Juxtanuclear-localized aggregate. The nucleus was visualized by DAPI staining. Values are calculated from 100–200 cells. (Related to Figure 5).
(TIF)

Figure S6 3D-SIM images of Hsp104Y662A-GFP-harboring cells stained with Alexa fluor 568 Phalloidin. Blue arrows indicate where Hsp104-associated aggregates (green: Hsp104Y662A-GFP) are lining up along actin cables (red: Alexa fluor 568 phalloidin) and the zoomed region shows that in some instances aggregates are wrapping around the cable (yellow arrows). Scale bars in the whole cell image  = 0.5 µm, scale bars in zoomed images  = 0.2 µm. (Related to Figure 6 C, D).
(TIF)

Table S1 Genotypes and sources of yeast strains used in this study (related to Materials and Methods).
(DOCX)

Table S2 Genetic interactions identified in the *SIR2* ts SGA screen (related to Figure 3).
(DOCX)

Movie S1 3D-SIM Z-stack movie of protein aggregates and the actin cytoskeleton (related to Figure 6 C,D).
(AVI)
