# Supplementary figures and images for: Essential Genetic Interactors of SIR2 Required for Spatial Sequestration and Asymmetrical Inheritance of Protein Aggregates
Source: PLoS Genet. 2014 Jul 31;10(7):e1004539. doi: 10.1371/journal.pgen.1004539 (PMC4117435; doi:10.1371/journal.pgen.1004539)

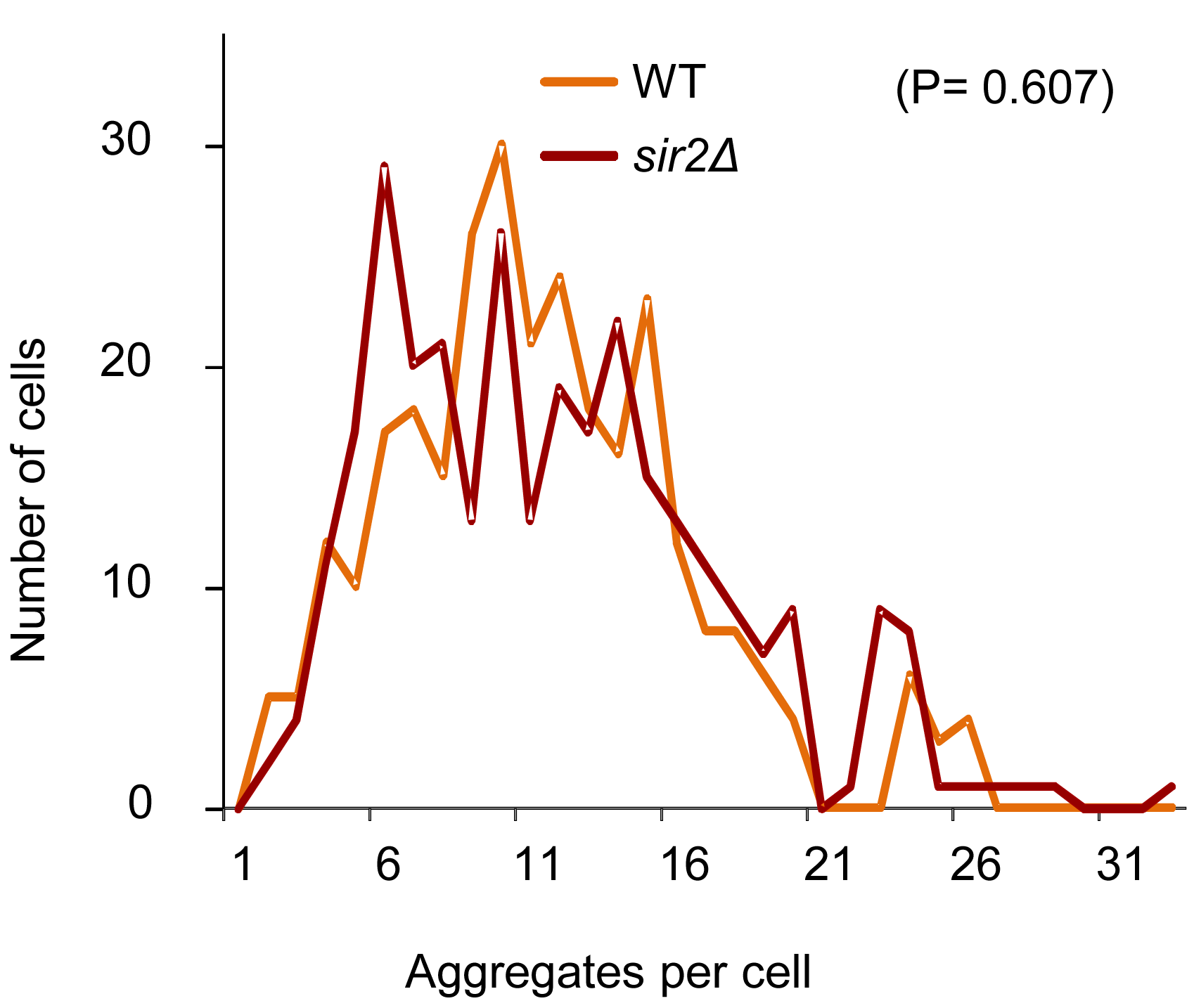

Supplement: Figure S1 — Distribution in the number of protein aggregates (Hsp104-GFP) per cell in wild type (orange) and sir2Δ (red) populations. The average number of aggregates per cell was 11.88 and 11.4 in the wild type and sir2Δ mutant, respectively. Average values are calculated from 300–400 cells. The statistical significance of observed differences was determined with the two-tailed U-test. P-values are indicated in the figure. (Related to Figure 2E). (TIF) [file pgen.1004539.s001.tif]

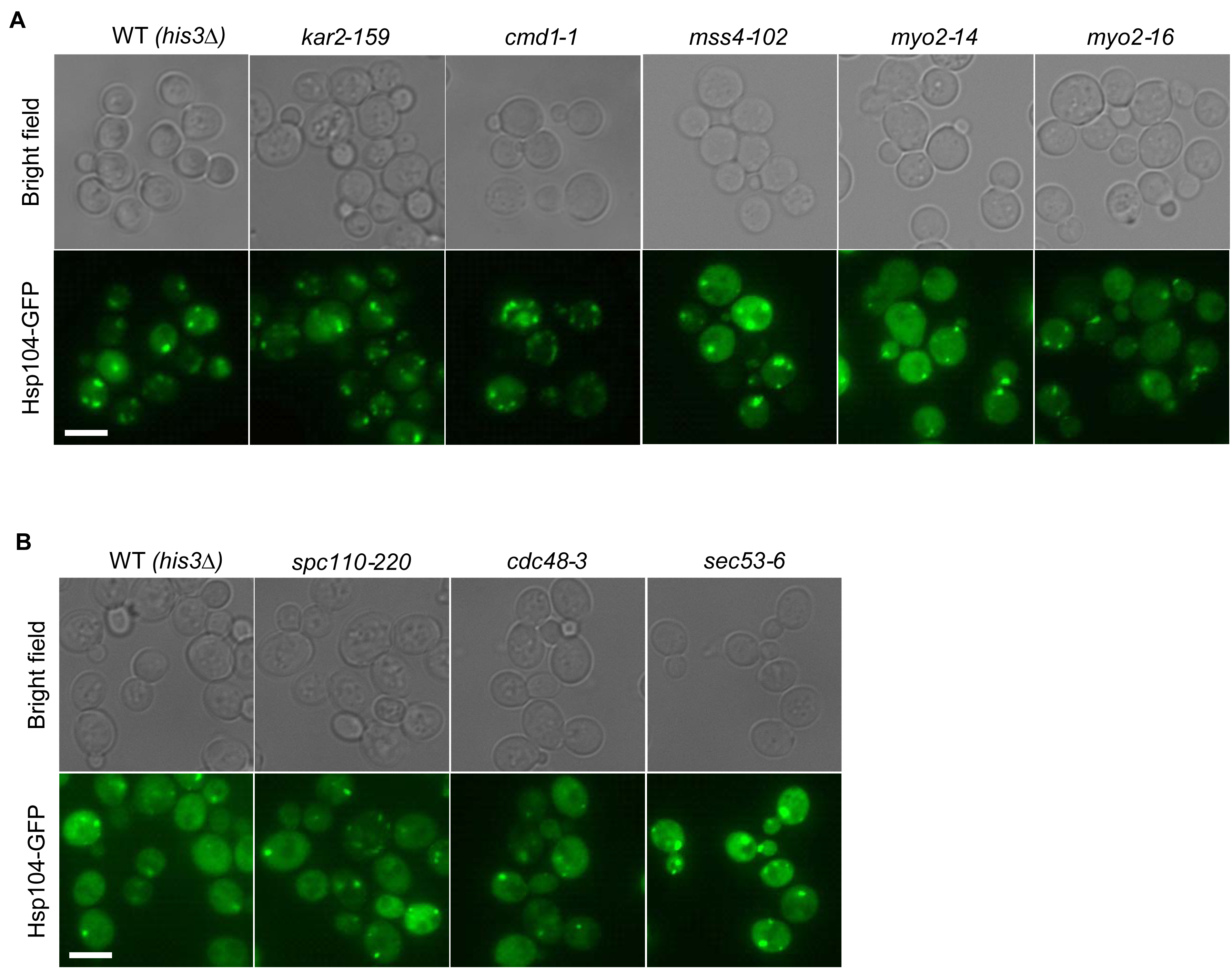

Supplement: Figure S2 — Representative images showing Hsp104-GFP aggregates (bottom panel) in the wild type (his3Δ) and ts mutant cells tested. A&B. Mutants were tested in 2 separate sets of experiments. Bright field images are showed in the upper panel. Scale bar = 5 µm. (Related to Figure 4). (TIF) [file pgen.1004539.s002.tif]

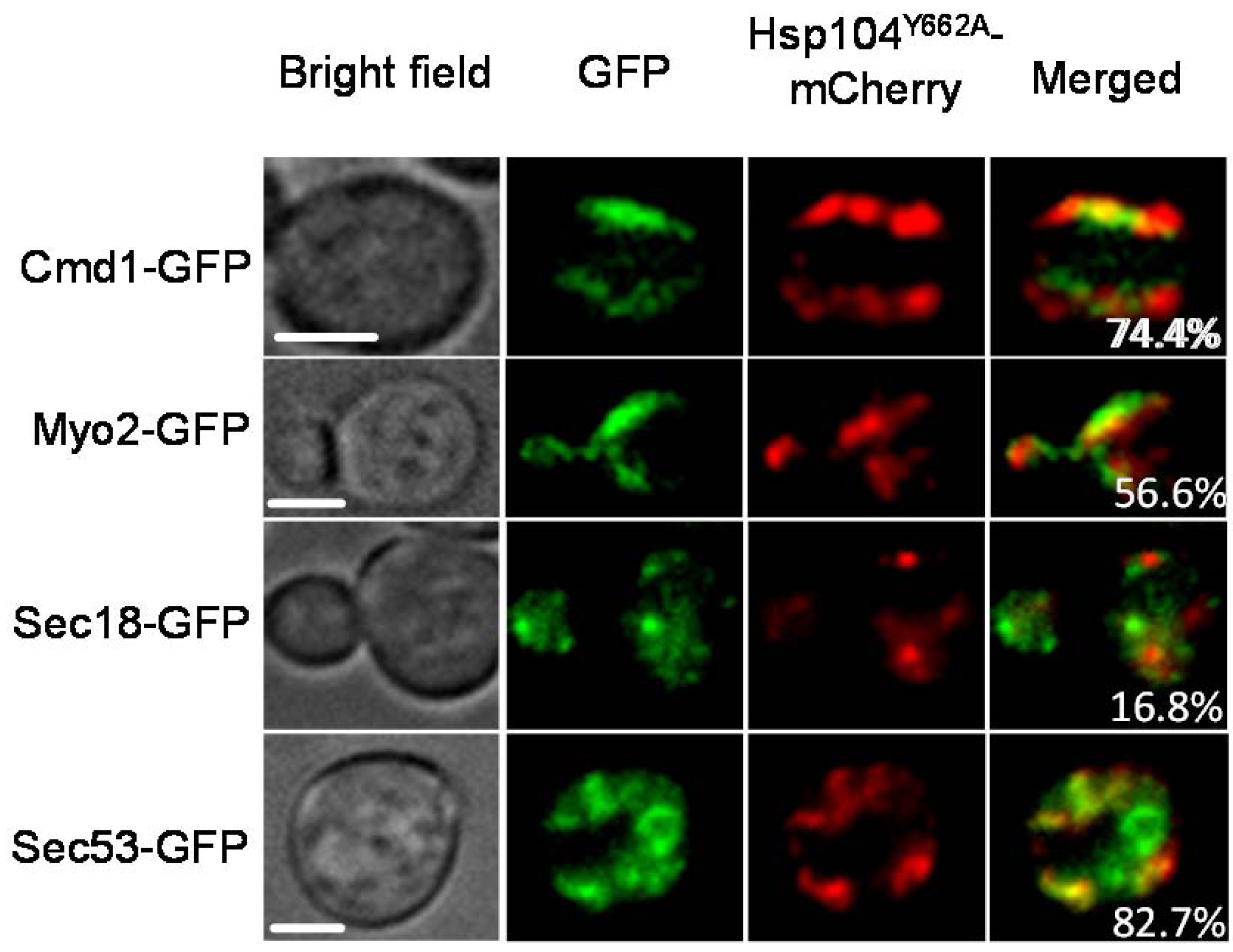

Supplement: Figure S3 — Hsp104Y662A-mCherry aggregates co-localize with some proteins encoded by essential genes required for asymmetrical aggregate segregation. As shown, some Cmd1-GFP (74.4%, tope panel) or Myo2-GFP (56.6%, second panel from top) structures co-localize with Hsp104Y662A-mCherry aggregates. The co-localization of Sec18-GFP and Hsp104Y662A-mCherry aggregates can be observed in only about 16.8% of cells (third panel). Sec53-GFP structures overlaps with some Hsp104Y662A-mCherry aggregates (82.7%). Scale bar = 5 µm. (Related to Figure 5). (TIF) [file pgen.1004539.s003.tif]

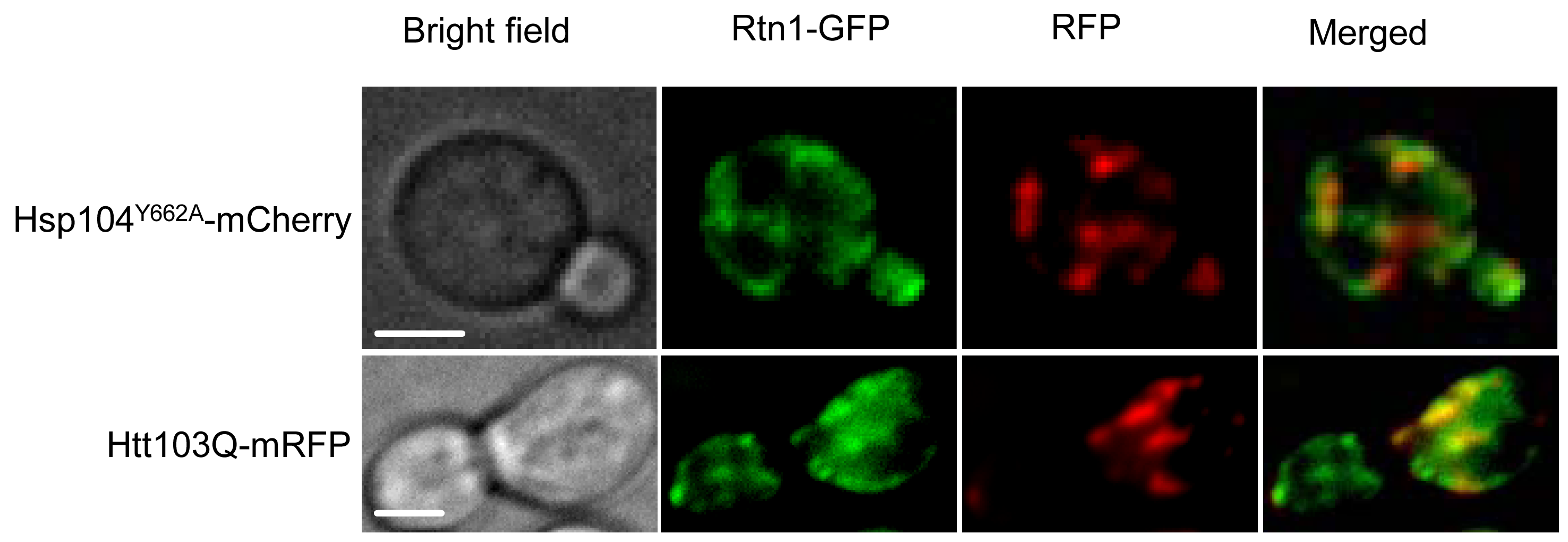

Supplement: Figure S4 — Cortical and cytoplasmic Rtn1-GFP signals show partial overlap with Hsp104Y662A-mCherry and Htt103Q-mRFP aggregates. Scale bar = 5 µm. (Related to Figure 5). (TIF) [file pgen.1004539.s004.tif]

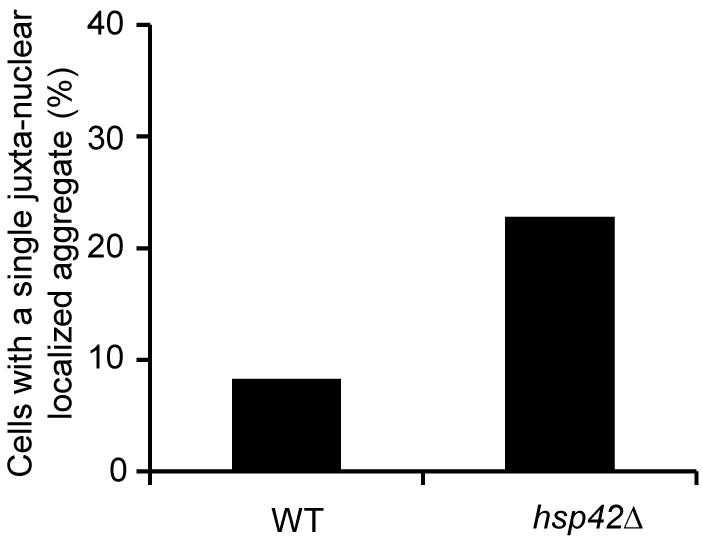

Supplement: Figure S5 — The hsp42Δ mutant displays increased number of cells with a single Juxtanuclear-localized aggregate. The nucleus was visualized by DAPI staining. Values are calculated from 100–200 cells. (Related to Figure 5). (TIF) [file pgen.1004539.s005.tif]

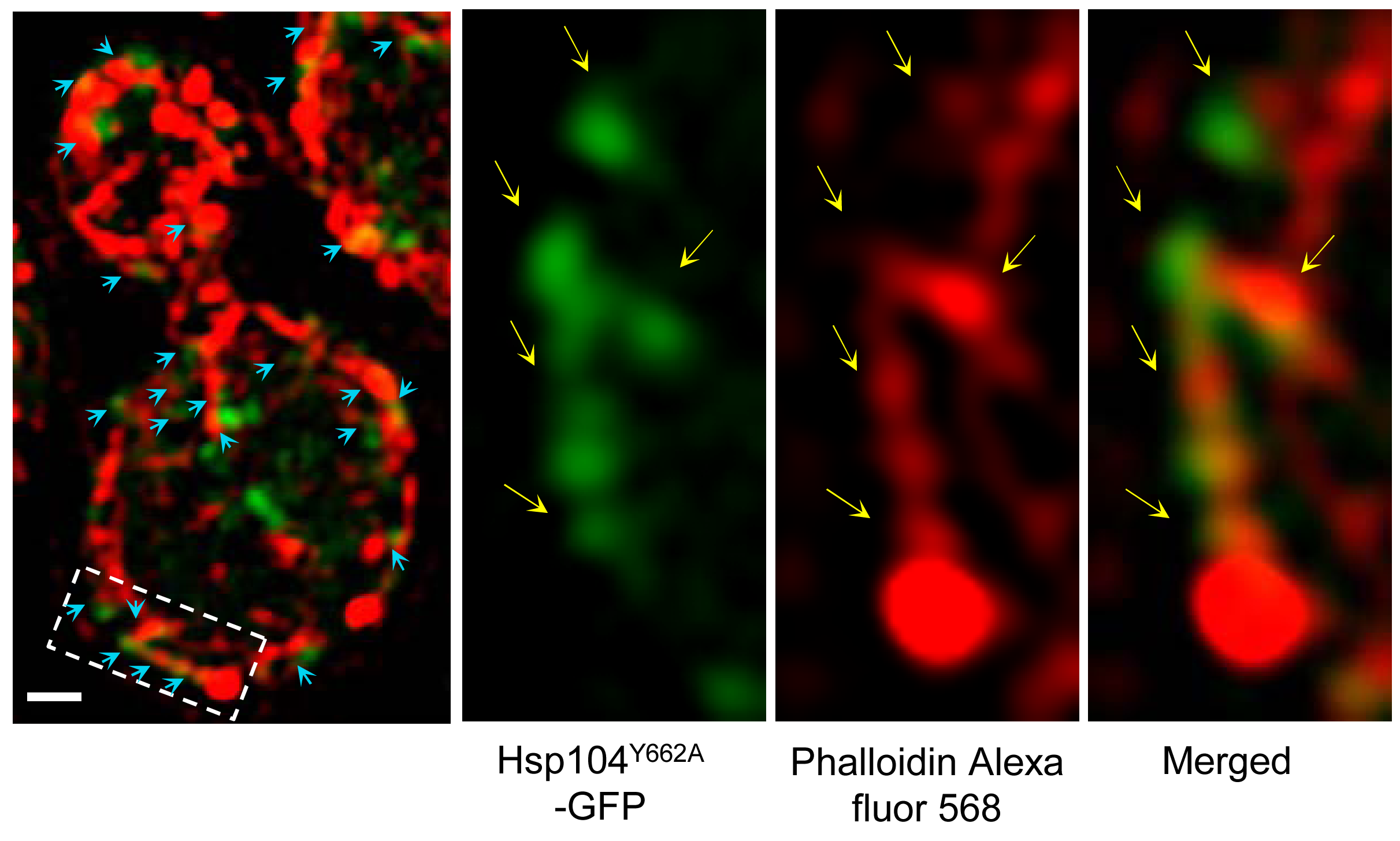

Supplement: Figure S6 — 3D-SIM images of Hsp104Y662A-GFP-harboring cells stained with Alexa fluor 568 Phalloidin. Blue arrows indicate where Hsp104-associated aggregates (green: Hsp104Y662A-GFP) are lining up along actin cables (red: Alexa fluor 568 phalloidin) and the zoomed region shows that in some instances aggregates are wrapping around the cable (yellow arrows). Scale bars in the whole cell image = 0.5 µm, scale bars in zoomed images = 0.2 µm. (Related to Figure 6 C, D). (TIF) [file pgen.1004539.s006.tif]
