# Supplementary material for: Essential Genetic Interactors of SIR2 Required for Spatial Sequestration and Asymmetrical Inheritance of Protein Aggregates
Source: PLoS Genet. 2014 Jul 31;10(7):e1004539. doi: 10.1371/journal.pgen.1004539 (PMC4117435; doi:10.1371/journal.pgen.1004539)
Supplement: Table S1 — Genotypes and sources of yeast strains used in this study (related to Materials and Methods). (DOCX) [file pgen.1004539.s007.docx]

**Table S1.** Genotypes and sources of yeast strains used in this study

|  | NAME | GENOTYPE | SOURCE | COMMENT |
| --- | --- | --- | --- | --- |
| 1 | BY4741 | *MAT*a *his3∆1 leu2∆0 met15∆0 ura3∆0* | EUROSCARF |  |
| 2 | sir2∆ | BY4741 *sir2∆::kanMX4* | EUROSCARF |  |
| 3 | WT mHtt103Q | BY4741 *pYES2-mHtt103Q-GFP* | This study | Plasmid from Michael Y. Sherman lab. |
| 4 | sir2 mHtt103Q | BY4741 *sir2∆::kanMX4 pYES2-mHtt103Q-GFP* | This study |  |
| 5 | WT Shs1-GFP | BY4741 *SHS1::GFP-HISMX6* | Invitrogen |  |
| 6 | sir2 Shs1-GFP | BY4741 *sir2∆::kanMX4 Shs1::GFP-HISMX6* | This study |  |
| 7 | Hsp104-GFP | BY4741 *HSP104::GFP-HISMX6* | Invitrogen |  |
| 8 | sir2∆ Hsp104-GFP | BY4741 *sir2∆::URA3 HSP104::GFP-HISMX6* | This lab |  |
| 9 | Y7092 | *MAT*α *can1*Δ*::STE2pr-Sp_his5 lyp1*Δ *ura3*Δ*0 leu2*Δ*0 his3*Δ*1 met15*Δ*0 LYS2+* | Boone Lab | Parental strain for SGA query |
| 10 | Ycrl | Y7092 *his3Δ::natMX4 hmr***a***1-hmr***a***2Δ::URA3 hmlα1-hmlα2Δ::hphR* | This lab | SGA query strain for control set |
| 11 | Ysir2-srl | Y7092 *sir2Δ::natMX4 hmr***a***1-hmr***a***2Δ::URA3 hmlα1-hmlα2Δ::hphR* | This lab | SGA query strain |
| 12 | Hsp104-GFP LEU2 | Y7092 *HSP104::GFP-LEU2* | This lab |  |
| 13 | cct1-1 | BY4741 *cct1-2::kanMX4* | Boone Lab |  |
| 14 | sir2 cct1-2 | BY4741 *sir2Δ::natMX4 cct1-2::kanMX4* | This study |  |
| 15 | cmd1-1 | BY4741 *cct1-2::kanMX4* | Boone Lab |  |
| 16 | sir2 cmd1-1 | BY4741 *sir2Δ::natMX4 cct1-2::kanMX4* | This study |  |
| 17 | his3 Hsp104-GFP | *MATa his3Δ::kanMX4 HSP104::GFP-LEU2 can1Δ::STE2pr-Sp_his5 lyp1Δ ura3Δ0 leu2Δ0 met15Δ0 LYS2+* | This lab |  |
| 18 | cmd1-1 Hsp104-GFP | *MATa cmd1-1::kanMX4 HSP104::GFP-LEU2 can1Δ::STE2pr-Sp_his5 lyp1Δ ura3Δ0 leu2Δ0 his3Δ1 met15Δ0 LYS2+* | This study |  |
| 19 | mss4-102 Hsp104-GFP | *MATa mss4-102::kanMX4 HSP104::GFP-LEU2 can1Δ::STE2pr-Sp_his5 lyp1Δ ura3Δ0 leu2Δ0 his3Δ1 met15Δ0 LYS2+* | This study |  |
| 20 | myo2-14 Hsp104-GFP | *MATa myo2-14::kanMX4 HSP104::GFP-LEU2 can1Δ::STE2pr-Sp_his5 lyp1Δ ura3Δ0 leu2Δ0 his3Δ1 met15Δ0 LYS2+* | This study |  |
| 21 | scd5-PP1D2 Hsp104-GFP | *MATa scd5-PP1D2::kanMX4 HSP104::GFP-LEU2 can1Δ::STE2pr-Sp_his5 lyp1Δ ura3Δ0 leu2Δ0 his3Δ1 met15Δ0 LYS2+* | This study |  |
| 22 | act1-133 Hsp104-GFP | *MATa act1-133::kanMX4 HSP104::GFP-LEU2 can1Δ::STE2pr-Sp_his5 lyp1Δ ura3Δ0 leu2Δ0 his3Δ1 met15Δ0 LYS2+* | This study |  |
| 23 | rho3-1 Hsp104-GFP | *MATa rho3-1::kanMX4 HSP104::GFP-LEU2 can1Δ::STE2pr-Sp_his5 lyp1Δ ura3Δ0 leu2Δ0 his3Δ1 met15Δ0 LYS2+* | This study |  |
| 24 | myo2-16 Hsp104-GFP | *MATa myo2-16::kanMX4 HSP104::GFP-LEU2 can1Δ::STE2pr-Sp_his5 lyp1Δ ura3Δ0 leu2Δ0 his3Δ1 met15Δ0 LYS2+* | This study |  |
| 25 | spc110-220 Hsp104-GFP | *MATa spc110-220::kanMX4 HSP104::GFP-LEU2 can1Δ::STE2pr-Sp_his5 lyp1Δ ura3Δ0 leu2Δ0 his3Δ1 met15Δ0 LYS2+* | This study |  |
| 26 | sec53-6 Hsp104-GFP | *MATa sec53-6::kanMX4 HSP104::GFP-LEU2 can1Δ::STE2pr-Sp_his5 lyp1Δ ura3Δ0 leu2Δ0 his3Δ1 met15Δ0 LYS2+* | This study |  |
| 27 | cdc48-3 Hsp104-GFP | *MATa cdc48-3::kanMX4 HSP104::GFP-LEU2 can1Δ::STE2pr-Sp_his5 lyp1Δ ura3Δ0 leu2Δ0 his3Δ1 met15Δ0 LYS2+* | This study |  |
| 28 | vct2 Hsp104-GFP | *MATa vct2::kanMX4 HSP104::GFP-LEU2 can1Δ::STE2pr-Sp_his5 lyp1Δ ura3Δ0 leu2Δ0 his3Δ1 met15Δ0 LYS2+* | This study |  |
| 29 | vct3 Hsp104-GFP | *MATa vct3::kanMX4 HSP104::GFP-LEU2 can1Δ::STE2pr-Sp_his5 lyp1Δ ura3Δ0 leu2Δ0 his3Δ1 met15Δ0 LYS2+* | This study |  |
| 30 | kar2-159 Hsp104-GFP | *MATa kar2-159::kanMX4 HSP104::GFP-LEU2 can1Δ::STE2pr-Sp_his5 lyp1Δ ura3Δ0 leu2Δ0 his3Δ1 met15Δ0 LYS2+* | This study |  |
| 31 | sec7-1 Hsp104-GFP | *MATa sec7-1::kanMX4 HSP104::GFP-LEU2 can1Δ::STE2pr-Sp_his5 lyp1Δ ura3Δ0 leu2Δ0 his3Δ1 met15Δ0 LYS2+* | This study |  |
| 32 | sec8-9 Hsp104-GFP | *MATa sec8-9::kanMX4 HSP104::GFP-LEU2 can1Δ::STE2pr-Sp_his5 lyp1Δ ura3Δ0 leu2Δ0 his3Δ1 met15Δ0 LYS2+* | This study |  |
| 33 | sec11-2 Hsp104-GFP | *MATa sec11-2::kanMX4 HSP104::GFP-LEU2 can1Δ::STE2pr-Sp_his5 lyp1Δ ura3Δ0 leu2Δ0 his3Δ1 met15Δ0 LYS2+* | This study |  |
| 34 | sec18-1 Hsp104-GFP | *MATa sec18-1::kanMX4 HSP104::GFP-LEU2can1Δ::STE2pr-Sp_his5 lyp1Δ ura3Δ0 leu2Δ0 his3Δ1 met15Δ0 LYS2+* | This study |  |
| 35 | sec20-1 Hsp104-GFP | *MATa sec20-1::kanMX4 HSP104::GFP-LEU2 can1Δ::STE2pr-Sp_his5 lyp1Δ ura3Δ0 leu2Δ0 his3Δ1 met15Δ0 LYS2+* | This study |  |
| 36 | sec22-3 Hsp104-GFP | *MATa sec22-3::kanMX4 HSP104::GFP-LEU2 can1Δ::STE2pr-Sp_his5 lyp1Δ ura3Δ0 leu2Δ0 his3Δ1 met15Δ0 LYS2+* | This study |  |
| 37 | sec23-1 Hsp104-GFP | *MATa sec23-1::kanMX4 HSP104::GFP-LEU2 can1Δ::STE2pr-Sp_his5 lyp1Δ ura3Δ0 leu2Δ0 his3Δ1 met15Δ0 LYS2+* | This study |  |
| 38 | cct1-2 Hsp104-GFP | *MATa cct1-2::kanMX4 HSP104::GFP-LEU2 can1Δ::STE2pr-Sp_his5 lyp1Δ ura3Δ0 leu2Δ0 his3Δ1 met15Δ0 LYS2+* | This study |  |
| 39 | cct6-18 Hsp104-GFP | *MATa cct6-18::kanMX4 HSP104::GFP-LEU2 can1Δ::STE2pr-Sp_his5 lyp1Δ ura3Δ0 leu2Δ0 his3Δ1 met15Δ0 LYS2+* | This study |  |
| 40 | cdc5-1 Hsp104-GFP | *MATa cdc5-1::kanMX4 HSP104::GFP-LEU2 can1Δ::STE2pr-Sp_his5 lyp1Δ ura3Δ0 leu2Δ0 his3Δ1 met15Δ0 LYS2+* | This study |  |
| 41 | kap95-L63A Hsp104-GFP | *MATa kap95-L63A::kanMX4 HSP104::GFP-LEU2 can1Δ::STE2pr-Sp_his5 lyp1Δ ura3Δ0 leu2Δ0 his3Δ1 met15Δ0 LYS2+* | This study |  |
| 42 | nup57-E17 Hsp104-GFP | *MATa nup57-E17::kanMX4 HSP104::GFP-LEU2 can1Δ::STE2pr-Sp_his5 lyp1Δ ura3Δ0 leu2Δ0 his3Δ1 met15Δ0 LYS2+* | This study |  |
| 43 | nsl1-5 Hsp104-GFP | *MATa nsl1-5::kanMX4 HSP104::GFP-LEU2 can1Δ::STE2pr-Sp_his5 lyp1Δ ura3Δ0 leu2Δ0 his3Δ1 met15Δ0 LYS2+* | This study |  |
| 44 | rfc4-20 Hsp104-GFP | *MATa rfc4-20::kanMX4 HSP104::GFP-LEU2 can1Δ::STE2pr-Sp_his5 lyp1Δ ura3Δ0 leu2Δ0 his3Δ1 met15Δ0 LYS2+* | This study |  |
| 45 | smc4-1 Hsp104-GFP | *MATa smc4-1::kanMX4 HSP104::GFP-LEU2 can1Δ::STE2pr-Sp_his5 lyp1Δ ura3Δ0 leu2Δ0 his3Δ1 met15Δ0 LYS2+* | This study |  |
| 46 | tfc1-E447K Hsp104-GFP | *MATa tfc1-E447K::kanMX4 HSP104::GFP-LEU2 can1Δ::STE2pr-Sp_his5 lyp1Δ ura3Δ0 leu2Δ0 his3Δ1 met15Δ0 LYS2+* | This study |  |
| 47 | nse4-ts Hsp104-GFP | *MATa nse4-ts::kanMX4 HSP104::GFP-LEU2 can1Δ::STE2pr-Sp_his5 lyp1Δ ura3Δ0 leu2Δ0 his3Δ1 met15Δ0 LYS2+* | This study |  |
| 48 | mps3-1 Hsp104-GFP | *MATa mps3-1::kanMX4 HSP104::GFP-LEU2 can1Δ::STE2pr-Sp_his5 lyp1Δ ura3Δ0 leu2Δ0 his3Δ1 met15Δ0 LYS2+* | This study |  |
| 49 | smc2-8 Hsp104-GFP | *MATa smc2-8::kanMX4 HSP104::GFP-LEU2 can1Δ::STE2pr-Sp_his5 lyp1Δ ura3Δ0 leu2Δ0 his3Δ1 met15Δ0 LYS2+* | This study |  |
| 50 | tub4-Y445D Hsp104-GFP | *MATa tub4-Y445D::kanMX4 HSP104::GFP-LEU2 can1Δ::STE2pr-Sp_his5 lyp1Δ ura3Δ0 leu2Δ0 his3Δ1 met15Δ0 LYS2+* | This study |  |
| 51 | cks1-35 Hsp104-GFP | *MATa cks1-35::kanMX4 HSP104::GFP-LEU2 can1Δ::STE2pr-Sp_his5 lyp1Δ ura3Δ0 leu2Δ0 his3Δ1 met15Δ0 LYS2+* | This study |  |
| 52 | cdc23-1 Hsp104-GFP | *MATa cdc23-1::kanMX4 HSP104::GFP-LEU2 can1Δ::STE2pr-Sp_his5 lyp1Δ ura3Δ0 leu2Δ0 his3Δ1 met15Δ0 LYS2+* | This study |  |
| 53 | rpt1-1 Hsp104-GFP | *MATa rpt1-1::kanMX4 HSP104::GFP-LEU2 can1Δ::STE2pr-Sp_his5 lyp1Δ ura3Δ0 leu2Δ0 his3Δ1 met15Δ0 LYS2+* | This study |  |
| 54 | rpn1-82 Hsp104-GFP | *MATa rpn1-82 ::kanMX4 HSP104::GFP-LEU2 can1Δ::STE2pr-Sp_his5 lyp1Δ ura3Δ0 leu2Δ0 his3Δ1 met15Δ0 LYS2+* | This study |  |
| 55 | his3 pYES2-GFP | *MATa his3Δ::kanMX4 pYES2-GFP can1Δ::STE2pr-Sp_his5 lyp1Δ ura3Δ0 leu2Δ0 met15Δ0 LYS2+* | This study |  |
| 56 | cmd1-1 pYES2-GFP | *MATa cmd1-1::kanMX4 pYES2-GFP can1Δ::STE2pr-Sp_his5 lyp1Δ ura3Δ0 leu2Δ0 his3Δ1 met15Δ0 LYS2+* | This study |  |
| 57 | myo2-14 pYES2-GFP | *MATa myo2-14::kanMX4 pYES2-GFP can1Δ::STE2pr-Sp_his5 lyp1Δ ura3Δ0 leu2Δ0 his3Δ1 met15Δ0 LYS2+* | This study |  |
| 58 | sec53-6 pYES2-GFP | *MATa sec53-6::kanMX4 pYES2-GFP can1Δ::STE2pr-Sp_his5 lyp1Δ ura3Δ0 leu2Δ0 his3Δ1 met15Δ0 LYS2+* | This study |  |
| 59 | sec18-1 pYES2-GFP | *MATa sec18-1::kanMX4 pYES2-GFP can1Δ::STE2pr-Sp_his5 lyp1Δ ura3Δ0 leu2Δ0 his3Δ1 met15Δ0 LYS2+* | This study |  |
| 60 | his3 pYES2-mHtt103Q-GFP | *MATa his3Δ::kanMX4 pYES2-mHtt103Q-GFP can1Δ::STE2pr-Sp_his5 lyp1Δ ura3Δ0 leu2Δ0 met15Δ0 LYS2+* | This study |  |
| 61 | cmd1-1 pYES2-mHtt103Q-GFP | *MATa cmd1-1::kanMX4 pYES2-mHtt103Q-GFP can1Δ::STE2pr-Sp_his5 lyp1Δ ura3Δ0 leu2Δ0 his3Δ1 met15Δ0 LYS2+* | This study |  |
| 62 | myo2-14 pYES2-mHtt103Q-GFP | *MATa myo2-14::kanMX4 pYES2-mHtt103Q-GFP can1Δ::STE2pr-Sp_his5 lyp1Δ ura3Δ0 leu2Δ0 his3Δ1 met15Δ0 LYS2+* | This study |  |
| 63 | sec53-6 pYES2-mHtt103Q-GFP | *MATa sec53-6::kanMX4 pYES2-mHtt103Q-GFP can1Δ::STE2pr-Sp_his5 lyp1Δ ura3Δ0 leu2Δ0 his3Δ1 met15Δ0 LYS2+* | This study |  |
| 64 | sec18-1 pYES2-mHtt103Q-GFP | *MATa sec18-1::kanMX4 pYES2-mHtt103Q-GFP can1Δ::STE2pr-Sp_his5 lyp1Δ ura3Δ0 leu2Δ0 his3Δ1 met15Δ0 LYS2+* | This study |  |
| 65 | HSP104Y662A -mCherry | BY4741 *HSP104 Y662A-mCherry-hphMX4* | This lab |  |
| 66 | hsp42 HSP104 Y662A-GFP-HIS3 | BY4741 *hsp42∆::kanMX4 HSP104 Y662A-GFP-HIS3* | This study |  |
| 67 | Cmd1-GFP HSP104Y662A -mCherry | BY4741  *CMD1-GFP-HISMX6 HSP104 Y662A-mCherry-hphMX4* | This study |  |
| 68 | Myo2-GFP HSP104Y662A -mCherry | BY4741  *MYO2-GFP-HISMX6 HSP104 Y662A-mCherry-hphMX4* | This study |  |
| 69 | Sec18-GFP HSP104Y662A -mCherry | BY4741  *SEC18-GFP-HISMX6 HSP104 Y662A-mCherry-hphMX4* | This study |  |
| 70 | Sec53-GFP HSP104Y662A -mCherry | BY4741  *SEC53-GFP-HISMX6 HSP104 Y662A-mCherry-hphMX4* | This study |  |
| 71 | Rtn1-GFP HSP104Y662A -mCherry | BY4741  *RTN1-GFP-HISMX6 HSP104 Y662A-mCherry-hphMX4* | This study |  |
| 72 | hsp42 mHtt103Q | BY4741 *hsp42∆::kanMX4 pYES2-mHtt103Q-mRFP* | This study |  |
| 73 | Cmd1-GFP Htt103Q-mRFP | BY4741  *CMD1-GFP-HISMX6 pYES2-mHtt103Q-mRFP* | This study |  |
| 74 | Myo2-GFP Htt103Q-mRFP | BY4741  *MYO2-GFP-HISMX6 pYES2-mHtt103Q-mRFP* | This study |  |
| 75 | Sec18-GFP Htt103Q-mRFP | BY4741  *SEC18-GFP-HISMX6 pYES2-mHtt103Q-mRFP* | This study |  |
| 76 | Sec53-GFP Htt103Q-mRFP | BY4741  *SEC53-GFP-HISMX6 pYES2-mHtt103Q-mRFP* | This study |  |
| 77 | Rtn1-GFP Htt103Q-mRFP | BY4741  *RTN1-GFP-HISMX6 pYES2-mHtt103Q-mRFP* | This study |  |
| 78 | Hsp104-GFP Htt103Q-mRFP | BY4741  *HSP104-GFP-HISMX6 pYES2-mHtt103Q-mRFP* | This study |  |
| 79 | Rnq1-mRFP | BY4741  *pRS423-Cup1-Rnq1-mRFP* | This study | Plasmid from Dr. Douglas Cyr lab |
| 80 | ABP140-3GFP HSP104Y662A-mCherry | *BY4741 HSP104 Y662A-mCherry-hphMX4 ABP140-3GFP-LEU2* | This lab |  |
