# Supplementary material for: Essential Genetic Interactors of SIR2 Required for Spatial Sequestration and Asymmetrical Inheritance of Protein Aggregates
Source: PLoS Genet. 2014 Jul 31;10(7):e1004539. doi: 10.1371/journal.pgen.1004539 (PMC4117435; doi:10.1371/journal.pgen.1004539)
Supplement: Table S2 — Genetic interactions identified in the SIR2 ts SGA screen (related to Figure 3). (DOCX) [file pgen.1004539.s008.docx]

**Table S2.** Genetic interactions identified in the *SIR2* ts SGA screen

|  |  |  |  |  |  |  |
| --- | --- | --- | --- | --- | --- | --- |
|  |  |  | Increase on doubling time (h) | | |  |
| No. | Orf Name | Allele | 30℃ | 34℃ | 38℃ | Interaction |
| 1 | YHR164C | dna2-2 | 2,94 | 2,64 | 1,88 | SS |
| 2 | YOL094C | rfc4-20 | 1,02 | 1,00 | 0,51 | SS |
| 3 | YNL006W | lst8-6 | 0,94 | 0,35 | 1,98 | SS |
| 4 | YDR498C | sec20-1 | 0,80 | 0,61 | 0,13 | SS |
| 5 | YMR001C | cdc5-1 | 0,75 | 0,73 | 2,14 | SS |
| 6 | YLR268W | sec22-3 | 0,74 | 0,91 | 1,45 | SS |
| 7 | YFL045C | sec53-6 | 0,68 | 0 | n.d. | SS |
| 8 | YKL145W | rpt1-1 | 0,67 | n.d. | 1,89 | SS |
| 9 | YBR080C | sec18-1 | 0,59 | 0 | n.d. | SS |
| 10 | YKL021C | mak11-2 | 0,49 | 0,71 | 0,60 | SS |
| 11 | YJL203W | prp21-ts | 0,38 | 0 | 0 | SS |
| 12 | YKL172W | ebp2-1 | 0,36 | 0,61 | 0,32 | SS |
| 13 | YLR212C | tub4-Y445D | 0,28 | 1,70 | 0,88 | SS |
| 14 | YDR182W | cdc1-1 | 0,25 | 0,24 | 0 | SS |
| 15 | YJL019W | mps3-1 | 0,24 | 0,70 | 1,13 | SS |
| 16 | YOR329C | scd5-PP1D2 | 0,22 | 1,20 | 2,14 | SS |
| 17 | YLR212C | tub4-ΔDSY | 0,21 | 0,30 | 1,47 | SS |
| 18 | YIR022W | sec11-2 | 0,20 | 0 | 0 | SS |
| 19 | YLR086W | smc4-1 | 0,18 | 0 | 0,60 | SS |
| 20 | YHR166C | cdc23-1 | 0,17 | 0 | 0,10 | SS |
| 21 | YGL130W | ceg1-34 | 0,16 | 0,98 | n.d. | SS |
| 22 | YPR055W | sec8-9 | 0,09 | 0 | 0 | SS |
| 23 | YBR155W | cns1-1 | 0 | 2,27 | 1,32 | SS |
| 24 | YDR212W | cct1-2 | 0 | 1,61 | n.d. | SS |
| 25 | YBR193C | med8-51 | 0 | 0,81 | n.d. | SS |
| 26 | YDR170C | sec7-1 | 0 | 0,48 | 0 | SS |
| 27 | YOL139C | cdc33-E72G | 0 | 0,43 | n.d. | SS |
| 28 | YDR145W | taf12-9 | 0 | 0,34 | n.d. | SS |
| 29 | YML130C | ero1-1 | 0 | 0,22 | 0 | SS |
| 30 | YHR027C | rpn1-821 | 0 | 0,22 | 0,72 | SS |
| 31 | YOL021C | dis3-1 | 0 | 0,12 | 0,24 | SS |
| 32 | YGR119C | nup57-E17 | 0 | 0 | 2,05 | SS |
| 33 | YFL039C | act1-133 | 0 | 0 | 1,95 | SS |
| 34 | YDR356W | spc110-220 | 0 | 0 | 1,51 | SS |
| 35 | YFR031C | smc2-8 | 0 | 0 | 1,43 | SS |
| 36 | YBR135W | cks1-35 | 0 | 0 | 1,23 | SS |
| 37 | YDR188W | cct6-18 | 0 | 0 | 1,15 | SS |
| 38 | YPL228W | cet1-15 | 0 | 0 | 0,99 | SS |
| 39 | YBR109C | cmd1-1 | 0 | 0 | 0,46 | SS |
| 40 | YBR123C | tfc1-E447K | 0 | 0 | 0,44 | SS |
| 41 | YDL126C | cdc48-3 | 0 | 0 | 0,43 | SS |
| 42 | YLR103C | cdc45-27 | 0 | 0 | 0,41 | SS |
| 43 | YOL135C | med7-141 | 0 | 0 | 0,23 | SS |
| 44 | YOR244W | esa1-D414 | 0 | 0 | 0,23 | SS |
| 45 | YDL105W | nse4-ts1 | 0 | 0 | 0,21 | SS |
| 46 | YHR024C | mas2-10 | 0 | 0 | 0,19 | SS |
| 47 | YLR347C | kap95-L63A | 0 | 0 | 0,16 | SS |
| 48 | YNL216W | rap1-2 | 0 | 0 | 0,14 | SS |
| 49 | YPL228W | cet1-2 | 0 | 0 | 0,14 | SS |
| 50 | YPL233W | nsl1-5 | 0 | 0 | 0,10 | SS |
|  |  |  |  |  |  |  |
| SS: Synthetic Sick; n.d.: no data; 0: no interaction | | | |  |  |  |
|  |  |  |  |  |  |  |
